# High-throughput and accurate cross-scale 3D sensing with a meta-depth sensor

**DOI:** 10.1093/nsr/nwag524

**Published:** 2026-08-21

**Authors:** Tao Yu, Ning Li, Qingqian Lang, Zhexuan Cao, Yuhan Hao, Laiyu Zhu, Jiamin Wu, Qionghai Dai

**Affiliations:** Beijing National Research Center for Information Science and Technology, Tsinghua University, Beijing 100084, China; School of Electronic and Information Engineering, Zhengzhou University, Zhengzhou 450001, China; Beijing National Research Center for Information Science and Technology, Tsinghua University, Beijing 100084, China; Department of Automation, Tsinghua University, Beijing 100084, China; Institute for Brain and Cognitive Sciences, Tsinghua University, Beijing 100084, China; Department of Automation, Tsinghua University, Beijing 100084, China; Institute for Brain and Cognitive Sciences, Tsinghua University, Beijing 100084, China; Department of Automation, Tsinghua University, Beijing 100084, China; Institute for Brain and Cognitive Sciences, Tsinghua University, Beijing 100084, China; Beijing National Research Center for Information Science and Technology, Tsinghua University, Beijing 100084, China; Department of Automation, Tsinghua University, Beijing 100084, China; Institute for Brain and Cognitive Sciences, Tsinghua University, Beijing 100084, China; Beijing National Research Center for Information Science and Technology, Tsinghua University, Beijing 100084, China; Department of Automation, Tsinghua University, Beijing 100084, China; Institute for Brain and Cognitive Sciences, Tsinghua University, Beijing 100084, China

**Keywords:** depth sensing, light-field imaging, meta-imaging, foundation model

## Abstract

Depth sensing has been widely applied in industrial inspection, transportation and consumer electronics. However, existing methods still face an inherent trade-off between depth precision and data throughput. Here, we present a meta-imaging-empowered depth sensor (MDS), a fully passive and monocular modality, that transcends this limitation through aberration-robust disparity-depth calibration and vision-foundation-model-empowered fine-grained three-dimensional (3D) ranging based on a scanning light-field imaging system. Our system synergizes high-throughput disparity estimation and massive data priors from foundation models, enabling high-precision, fine-grained metric depth estimation across depth ranges from 0.1 to 500 m. Experimental validation demonstrates 2.5-mm absolute depth precision at a 1.5-m distance (0.18% relative error) and 3-megapixel resolution, outperforming conventional light-field depth sensors by 7 times in accuracy and 5 times in spatial resolution without sacrificing imaging speed with a virtual-scanning technique. We also achieve turbulence-robust remote depth sensing at the 500-m distance with a significantly denser spatial sampling rate compared with commercial LiDAR systems, and a depth estimation precision of 0.7 m at a 235-m distance. By unifying the throughput advantages of passive imaging with the high-precision depth estimation ability of physics-aware neural regression, the proposed MDS establishes a scalable paradigm for high-fidelity passive 3D sensing across scales, with applications ranging from industrial inspection to autonomous driving.

## Teaser

A passive and monocular depth sensor framework unifies high-throughput and accurate 3D imaging across scales from 0.1 m to 500 m.

## INTRODUCTION

Depth perception, the ability to spatially resolve three-dimensional (3D) structures, serves as a cornerstone of modern intelligent systems, ranging from autonomous navigation [1] requiring centimeter-level precision in dynamic environments to biomedical imaging [2] demanding micron-scale resolution for cellular analysis. While existing modalities excel in specific niches, they universally face a fundamental dichotomy: systems leveraging active illumination (e.g. LiDAR [3], time-of-flight [4]) achieve high precision at the cost of spatial sampling density, whereas passive methods (e.g. stereo vision [5], monocular imaging [6]) prioritize throughput (the number of pixels in the output depth map) but lack absolute depth accuracy. As a result, the need for depth-sensing technologies that simultaneously achieve high accuracy and high throughput has become a universal challenge across disciplines [7–13]. Resolving the fundamental conflict between throughput and accuracy in depth perception through a purely passive and monocular methodology has the potential to revolutionize three-dimensional perception across multi-scale scenarios ranging from industrial detection [14] to autonomous driving [15,16].

Depth-sensing technologies, whether active or passive, face an inherent physical trade-off between throughput and metric accuracy, further compounded by the fundamental challenge that any single principle struggles to provide robust depth perception across diverse scales. Active modalities—including LiDAR, time-of-flight (ToF) cameras and structured-light systems—achieve metrological rigor (subcentimetre to millimetre precision) through controlled illumination but face a fundamental energy-density trade-off. This severely limits sampling density: even advanced solid-state LiDAR systems (angular resolution has been improved to $0.01^\circ$–$0.04^\circ$ [3,17,18]) rarely exceed 0.02 megapixels [19], while ToF cameras are resolution-capped (*∼*1 megapixel) by infrared diffraction and SNR constraints [4]. Structured light enables dense reconstruction [20] but falters under occlusion, with metasurface [21] or color-conversion [22] enhancements still struggling in outdoor/long-range scenarios. Consequently, active systems compromise fine-scale geometry reconstruction, critically impairing tasks such as distant small-object detection in autonomous driving. Conversely, passive methods leverage ubiquitous complementary metal-oxide-semiconductor (CMOS) sensors to achieve high throughput (*> *10 megapixels) but suffer from metric inaccuracies rooted in information loss: stereo vision yields dense disparity maps yet requires textured surfaces [20] and struggles to resolve fine-grained occlusion due to an insufficient number of viewpoints; monocular depth estimation (e.g. via Monodepth2 [23] or Depth Anything [24] architectures) attains pixel-level resolution but lacks absolute scale without calibration; computational PSF techniques (defocus [25] or double-helix [26,27]) enable single-shot depth retrieval but contend with aliasing and ambiguity in macroscopic scenes. This dichotomy ultimately arises from core physical realities—active systems trade detection range against sampling density, while passive planar imaging intrinsically discards the angular/PSF information essential for unambiguous depth reconstruction from the 4D light field.

Light-field imaging addresses this by recording both spatial and angular light distributions through a microlens array (MLA) [28], theoretically enabling multi-view triangulation [29], depth-from-defocus [30] and epipolar geometry analysis [31]. However, conventional plenoptic cameras sacrifice spatial resolution for angular sampling [28], while multi-camera arrays [32] compensate the spatial resolution and angular resolution with a bulky and expensive structure. Prior works [33,34] established foundational advances in aberration-corrected light-field imaging, introducing the concept of meta-imaging—i.e. integrated scanning light-field (sLF) imaging—and demonstrating meta-imaging sensors capable of high-throughput, aberration-robust depth estimation. Nevertheless, scaling these systems to macroscopic distances remained hindered by lens- and turbulence-induced depth-disparity inconsistency and high-throughput absolute depth estimation bottlenecks in small physical baselines.

In this paper, we introduce a Meta-imaging-empowered computational Depth imaging Sensor (MDS) that overcomes the traditional precision-throughput trade-off through aberration-robust disparity-depth calibration and depth-vision-foundation-model-empowered fine-grained 3D ranging based on a scanning light-field imaging system. The system architecture combines a CMOS sensor with mask-coded MLA optics and a 2D galvo-scanning stage, enabling high-throughput subaperture image capture. System performance is enhanced through a joint aberration-disparity calibration approach that simultaneously addresses optical imperfections and depth-disparity inconsistency. We present a depth-vision-foundation-model-enhanced [24] light-field disparity estimation Mamba [35] network that establishes frequency-aware adaptive fusion mechanisms through an end-to-end learning paradigm, synergistically integrating holistic absolute depth perception of light-field multi-view imagery with the detail-preserving depth estimation capabilities of depth vision foundation models. This physics&data co-driven monocular passive metric depth estimation framework not only achieves millimeter-level depth resolution at 1.5 m but also delivers much denser depth measurements than LiDAR at 500-m ranges.

Experimental validation demonstrates MDS capabilities in high-throughput and high-precision metric depth estimation across multiple spatial scales (0.1–500 m). Monocular imaging achieves 2.5-mm absolute depth precision at a 1.5-m working distance with a resolution exceeding 3 megapixels—representing a five-fold spatial resolution enhancement over conventional light-field systems [28] while maintaining depth accuracy comparable to high-end structured-light cameras (Photoneo PhoXi 3D Scanner L; see Section S2 of the online supplementary material for more details). Under close-range working conditions (0.1 m), MDS achieves depth measurement of millimetre-scale microstructures with a mean error of 0.24 mm—representing a 10-fold improvement in precision over conventional light-field imaging. When deployed in long-range telescopic configurations (see Section S7), the MDS demonstrates turbulence-robust depth estimation at 500-m distances, achieving more detailed depth estimation with a significantly denser spatial sampling rate compared with commercial LiDAR systems. For moving targets, we also introduce a virtual-scanning modality [36] that enables camera-frame-rate depth estimation with high temporal continuity. This meta-imaging paradigm bridges the resolution advantages with quantitative metric depth estimation in a passive sensing manner, establishing new capabilities for large-scale 3D perception in complex environments. The integration of computational light-field imaging with a depth vision foundation model prior establishes a scalable framework for high-throughput, fine-grained metric depth estimation across spatial domains. The MDS enables the occlusion-free detection of fine structures and small targets, facilitating diverse industrial applications—such as measuring bumps and semiconductor bonding wires, or inspecting difficult-to-access pins—and supporting the long-range early detection of small objects in autonomous driving systems.

## RESULTS

### Principle of the MDS

Stereo vision systems enhance depth accuracy through extended baselines but introduce severe occlusion artifacts caused by view difference between the left and right cameras. On the other hand, conventional light-field imaging enables multi-view stereo vision through angular resolution enhancement at the cost of spatial resolution, inherently limiting subaperture image resolution. While capable of depth perception, traditional light-field imaging inherently suffers from limited physical baselines and small pixel-level disparities, precluding high-precision, high-throughput depth estimation. In our observation, enhancing pixel-level disparities without baseline extension is also effective for high-accuracy depth estimation under constrained baselines. This approach not only achieves comparable reconstruction fidelity but significantly mitigates occlusion challenges inherent to both active and passive stereo vision systems. Based on these principles, existing scanning light-field imaging techniques employ MLA-based microscanning to acquire high-throughput subaperture images [33]. This methodology amplifies pixel-level disparities without baseline modification, thereby enabling previously unattainable depth precision in compact optical configurations. Building on this foundation, MDS combines system-level aberration correction and disparity calibration with a high-performance disparity estimation algorithm empowered by a depth vision foundation model, establishing a computational depth imaging framework that achieves fine-grained absolute depth estimation. The MDS consists of a CMOS image sensor, mask-coded MLA, main lens, two-axis galvo-scanning mirror and control circuitry (Fig. 1a). The MLA is coated with a circular chromium mask around each lenslet. This circular aperture blocks light near the periphery of each microlens, thereby reducing the nulls in the optical transfer function that would otherwise cause artifacts during artifact-free reconstruction [33]. We implement lateral and longitudinal microscanning of incident light by positioning the 2D scanning mirror before the main lens, achieving synchronized scanning and camera triggering through control circuitry. The prototype system shown in Fig. 1b demonstrates a simple, reliable implementation of scanning light-field imaging. Further system integration could leverage high-speed piezo-stage scanning of either microlens arrays or CMOS modules [33], enabling more compact designs with reduced weight and power consumption.

**Figure 1.**
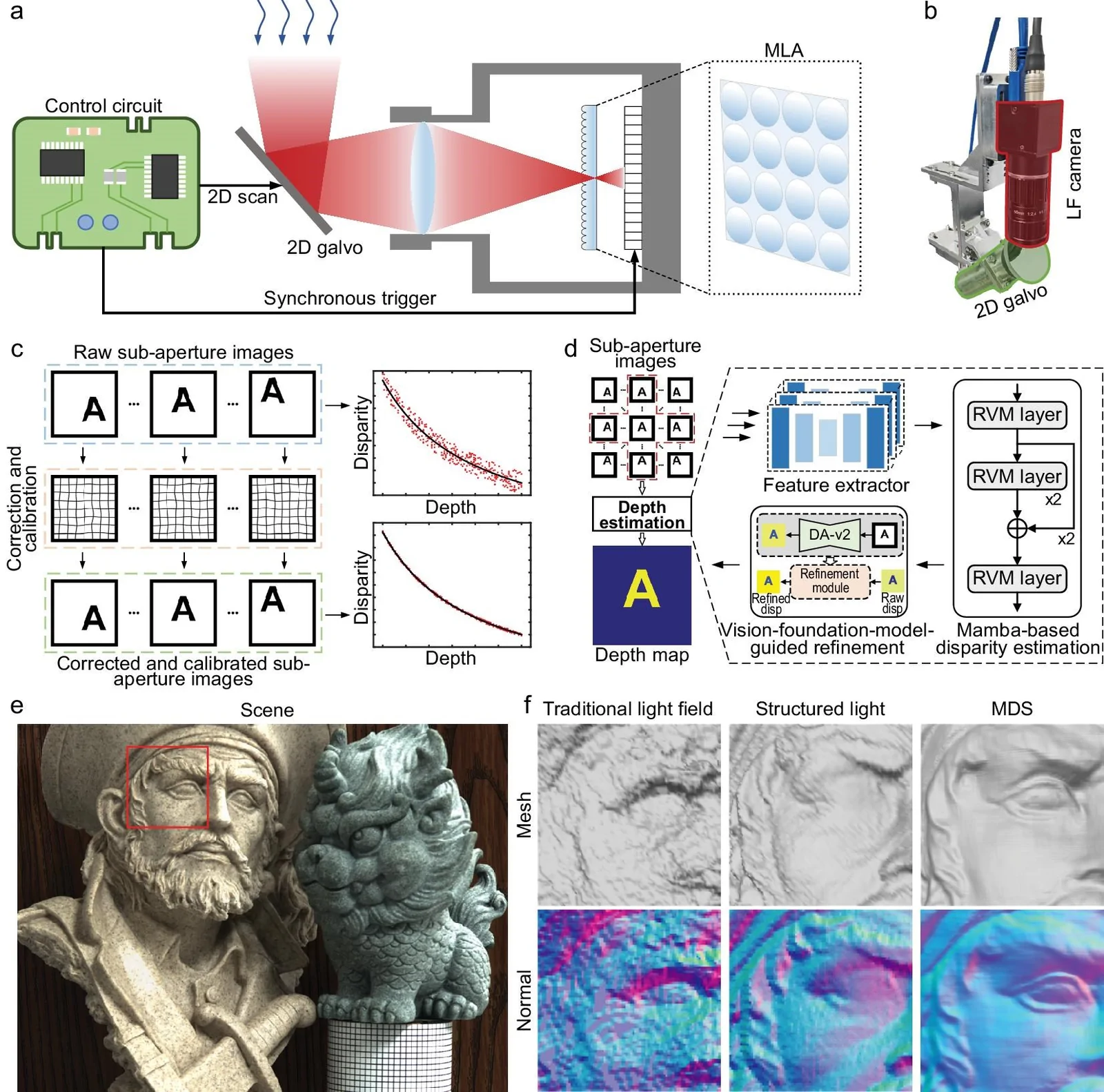
Principle of the MDS. (a) Schematic of the MDS. The MDS comprises four key components: (1) a micro-lens array for capturing multi-view images using a single complementary metal-oxide-semiconductor (CMOS) sensor, (2) a two-dimensional galvo mechanism to vibrate the mirror in front of the main lens for 2D scanning, (3) a control circuit to synchronize galvo scanning and image capture, and (4) a conventional CMOS sensor positioned at the back focal plane of the micro-lens array for high-throughput photon detection. (b) Prototype of the MDS. (c) System errors and optical aberrations lead to inconsistencies in the disparity-depth relationship, limiting depth estimation precision. To address this, a joint calibration and correction method is proposed, which corrects system aberrations and calibrates the disparity-depth relationship through a collaborative optimization framework. (d) Architecture of the depth vision foundation model (DA-v2, a vision foundation model for monocular depth estimation)-guided vision Mamba disparity estimation network (FMNet). This design leverages the large-scale data priors from depth vision foundational models and the physical priors of high-throughput multi-view measurements from scanning light-field (sLF) imaging to recover high-precision absolute depth with rich detail. (e) The test scene of statue group. (f) Comparison of magnified regions of mesh and normal results (highlighted by red rectangles in (e)) obtained from traditional light-field imaging, a high-precision structured-light camera and the proposed MDS.

Manufacturing and assembly imperfections inevitably introduce optical aberrations in any imaging system. These imperfections cause depth-disparity inconsistency and depth estimation ambiguity, fundamentally limiting high-precision depth measurement. Additionally, uncalibrated systems face challenges in establishing reliable depth-disparity relationships from nominal parameters, further constraining absolute depth estimation accuracy. To address these issues, we propose a joint aberration-disparity calibration algorithm (Fig. 1c) that simultaneously corrects optical aberrations and calibrates depth-disparity relationships within a unified framework through constraints on depth-disparity distribution consistency and subaperture image reprojection consistency (Fig. S2a). Conventional light-field aberration correction methods rely on multi-view consistency constraints at focal planes [33], which ignore the spatial correlation of aberrations within 3D measurement space and require strict optical axis alignment. Our method relaxes calibration data acquisition requirements by utilizing multi-plane calibration data across the 3D measurement space, improving aberration correction performance and depth-disparity calibration accuracy for precision 3D measurement. For long-range scenarios, robust spatial calibration can be achieved through co-registration of active depth measurements with key feature points in light-field images. The proposed calibration processing improves depth-disparity consistency by nearly five-fold and decreases depth estimation error by 34% (Fig. S2c and d), forming the foundation for high-precision metric depth estimation.

To achieve structurally coherent, detail-rich and accurate depth estimation from calibrated subaperture images, we developed a novel depth-vision-foundation-model-guided Mamba network (FMNet; Fig. 1d) for light-field disparity estimation. This framework synergistically integrates the subpixel-level physical disparity regression capability of Mamba networks [35] with the texture-agnostic depth recovery as well as the fine-grained detail reconstruction strengths of depth vision foundation models [24]. By systematically exploiting both triangulation-based geometric ranging information from multi-view light-field images and depth-correlated semantic cues from 2D imagery, the architecture uniquely bridges physics-driven measurement precision with data-driven contextual understanding. For pixel-wise disparity estimation, we employ a weight-shared feature extraction network across subaperture images to construct the matching cost volume. Unlike traditional 3D convolutional networks [29] that are computationally intensive, we implement a Mamba-based (state space model) [35] disparity regression network (Section S3) to extract deep semantic features and establish long-range spatial dependencies, enabling more efficient and accurate disparity estimation (Fig. S3d). Furthermore, passive depth estimation through stereo matching inherently suffers from texture-dependent ambiguity and poor detail reconstruction. To address this, we integrate depth vision foundation models like Depth Anything v2 (DA-v2)[24]—trained on large-scale synthetic data—to extract relative depth information from 2D images. While DA-v2 maintains scale ambiguity that prevents absolute depth measurement, it consistently provides detailed relative depth estimation even under texture-deficient conditions. We develop a foundation-model-guided disparity refinement module (Fig. 1d and Fig. S3c) to combine DA-v2’s detail reconstruction capability with sLF’s absolute depth estimation strength by establishing frequency-aware adaptive fusion mechanisms through an end-to-end learning paradigm. Our depth estimation framework establishes a unified two-paradigm fusion architecture, achieving high-precision, fine-grained metric depth estimation. This hybrid approach enables finer depth estimation than pure sLF and more accurate absolute measurement than standalone vision foundation models (Fig. S6). With the assistance of the vision foundation model, FMNet can not only effectively handle depth estimation in weak-texture scenes (Section S3 and Fig. S4) and albedo-varying smooth planes (Section S3 and Fig. S5) but also readily generalize to more challenging scenarios such as autonomous driving (Section S5 and Fig. S12).

As a computational depth imaging sensor, MDS achieves significant improvements in both depth estimation accuracy and throughput over conventional light-field imaging by combining system-level aberration-disparity joint calibration and intelligent disparity estimation algorithms. Results (Fig. S7) from testing traditional light-field depth estimation algorithms on scanning light-field data show that simply increasing subaperture image resolution through scanning can improve depth estimation performance to some extent, but a substantial gap remains compared with our proposed MDS method. This confirms the effectiveness and advancement of the proposed algorithm. Compared to high-end structured-light cameras, the MDS demonstrates superior depth estimation granularity, particularly in capturing surface normal variations (Fig. 1e and f) and occlusion-free detailed depth (Fig. 2g and h).

**Figure 2.**
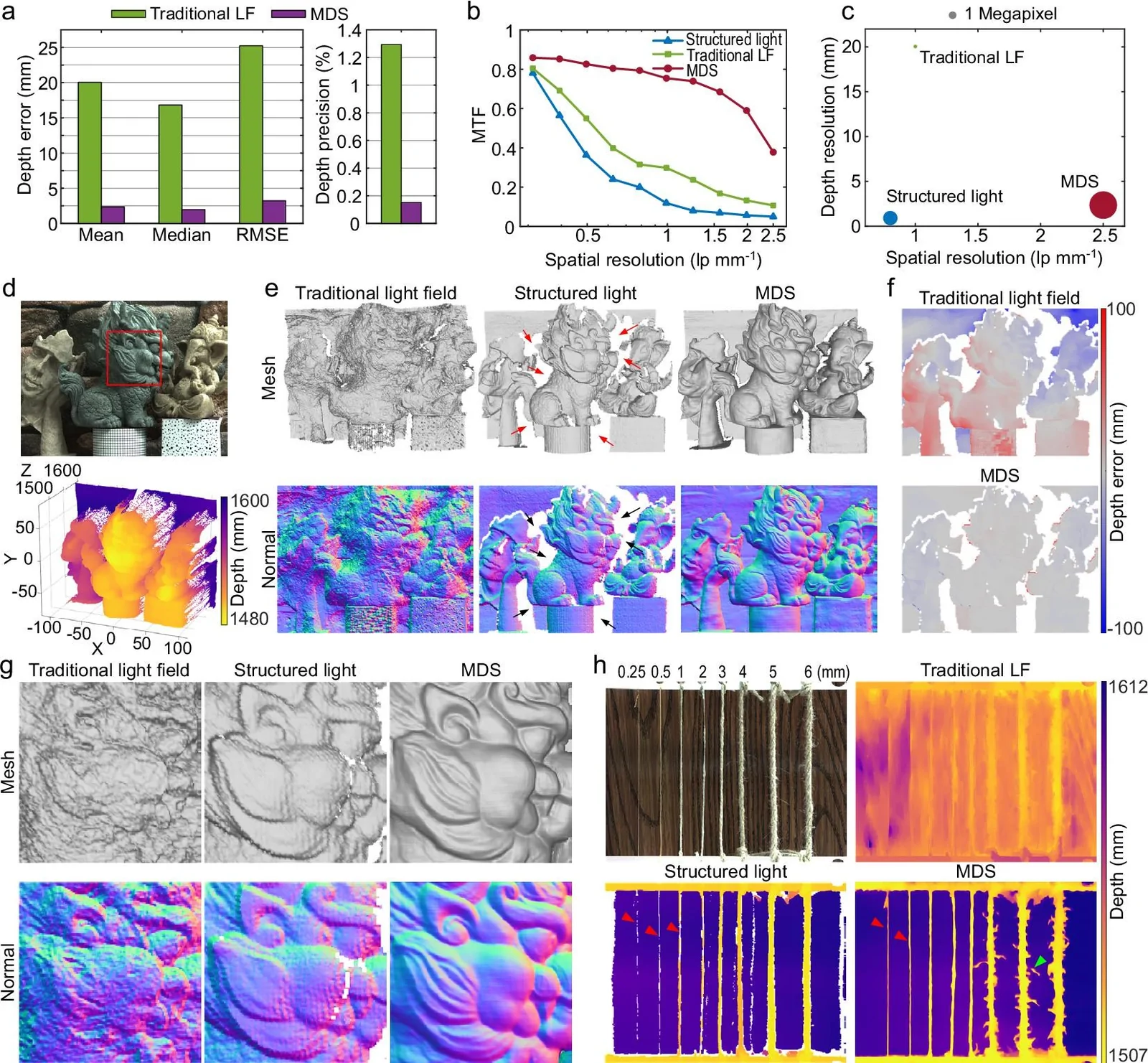
High-throughput and high-precision fine-grained depth perception with the MDS. (a) Depth estimation error and precision of traditional light-field imaging versus MDS. (b) MTF curves for the structured-light camera, tradition light-field imaging and MDS. (c) Comparison of overall depth estimation performance across different modalities, including depth resolution, spatial resolution and imaging throughput. (d) The test scene and the corresponding estimated point clouds. (e) Mesh and normal results obtained by traditional light-field imaging, a structured-light camera and MDS. (f) Comparison of depth estimation error maps between traditional light-field imaging and MDS. (g) Magnified regions of mesh and normal results (highlighted by red rectangles in (d)) obtained by traditional light-field imaging, a structured-light camera and MDS. (h) Depth estimation performance for fine structures. Experimental setup featuring hemp ropes with diameters ranging from 0.25 to 6 mm positioned at 1.5 m. Comparative depth estimation results of fine structures using MDS, conventional light-field imaging and structured-light imaging are provided. The MDS achieves complete and continuous reconstruction of 0.25-mm-diameter ropes, whereas structured-light imaging only sporadically resolves 1-mm-diameter ropes, and conventional light-field imaging fails to distinguish rope structures from background textures. Furthermore, the MDS reconstructs even finer microfibers (diameters *< *0.25 mm) surrounding the ropes, demonstrating its superior resolution for submillimeter structural reconstruction. This capability highlights the potential of the MDS for critical applications in unmanned systems (e.g. drones) to detect and avoid imperceptibly fine structures in extreme scenarios.

### High-accuracy and high-throughput depth estimation

The MDS enables multi-scale high-throughput and high-precision reconstruction; here we perform a comprehensive quantitative evaluation of near-field performance metrics (at 1.5 m), while extended analysis of long-range scenarios (300–500 m) is reserved for systematic benchmarking in subsequent sections. To validate the depth estimation advantages of the MDS, we conducted experimental evaluations focusing on reconstruction accuracy, spatial resolution and detail reconstruction performance. For depth accuracy assessment, we established 10 distinct test scenarios featuring static sculptural targets at a 1.5-m distance, with ground-truth depths acquired using a submillimeter-accurate structured-light camera (Section S2). Our depth estimation algorithm underwent joint optimization with both experimental and simulated training data (Sections S4 and S5). Comparative tests used conventional light-field imaging with central scanning position data (without joint aberration-disparity calibration) and existing depth estimation algorithms [37]. Experimental results demonstrate that the MDS achieves *≤*2.5-mm mean depth error at a 1.5-m working distance, corresponding to 0.18% relative accuracy—representing a seven-fold improvement over conventional light-field systems (Fig. 2a). Spatial resolution evaluation using USAF1951 resolution charts shows 5*×* enhancement compared to conventional light-field and structured-light imaging (Fig. 2b). The superior spatial resolution of the MDS enables depth perception of extremely fine structures—such as 0.25-mm-diameter ropes (or even thinner) at 1.5 m (Fig. 2h)—demonstrating its potential for critical applications in unmanned systems (e.g. drones) to detect and avoid imperceptibly fine structures in challenging operational scenarios. These results confirm the MDS’s dual capability in high-precision metric depth estimation and high spatial resolution, achieving over 3-megapixel imaging resolution for high-throughput depth perception (Fig. 2c).

We further analyzed depth detail reconstruction through mesh visualization and surface normal estimation. For the sculptural test target (Fig. 2d), our framework produces spatially continuous 3D reconstructions with rich surface details when integrated with vision foundation models (Fig. 2e), while maintaining minimal global depth error (Fig. 2f). Local magnification of the Qilin sculpture’s reconstructed mesh and surface normals demonstrates the MDS’s capability to resolve facial features that remain indistinguishable in both conventional light-field camera and structured-light imaging systems (Fig. 2g). Quantitative analysis shows that the MDS achieves ${< }17^\circ$ mean angular error in surface normal reconstruction (10% angular accuracy), doubling the performance of traditional light-field methods (Fig. S8d). This confirms the MDS’s capability to simultaneously capture high-precision depth information and surface normal variations.

Notably, while structured-light cameras achieve high depth accuracy through large baselines, this configuration introduces occlusion-induced depth voids (arrowed regions in Fig. 2e). The MDS overcomes this limitation through minimized baseline constraints from the primary lens configuration and enhanced disparity resolution via scanning light-field imaging, enabling complete scene depth estimation without occlusion artifacts.

### Multi-scale depth sensing

Multi-distance-scale depth perception plays critical roles across diverse domains, exemplified by the utility of close-range depth assessment in industrial inspection and the significance of long-range depth estimation in autonomous driving and remote sensing applications. However, passive imaging systems confront fundamental limitations in multi-scale depth perception: at close ranges, conventional stereo vision exhibits substantial near-field blind zones due to baseline constraints, while monocular imaging fails to provide reliable depth estimates. For practical long-range conditions, passive approaches are inherently compromised by turbulence-induced imaging aberrations, leading to measurement distortions and spatial inconsistency. The MDS demonstrates superior resolution in both lateral and axial dimensions, offering significant potential for multi-scale ranging and detection applications using the same paradigm.

We evaluated close-range depth perception performance using an MDS to image finely detailed 3D-printed structures positioned at approximately 10 cm (experimental details provided in Section S6). Pillars, thin plates and curved tubes (all features with a 1.2-mm diameter or thickness; Fig. 3a) were fabricated within a *30× 30*-mm$^2$ substrate area using high-precision 3D printing to simulate miniature industrial components. Utilizing the proposed MDS, we accurately reconstructed 3D point clouds of these microstructures (Fig. 3b), with reconstructions exhibiting superior fidelity to ground-truth geometry compared to conventional light-field imaging (Fig. 3c). Statistical analysis confirmed that the MDS delivers significantly more precise absolute height measurements for pillars and thin plates (Fig. 3d), achieving nearly an order-of-magnitude reduction in mean height estimation error versus conventional light-field methods (from 2.5 to 0.24 mm; Fig. 3e). For curved tubes, the MDS enabled more accurate spatial trajectory and 3D curvature quantification (Fig. 3f). Comparative experiments of the MDS without aberration correction (Fig. 3d–g) further validated the efficacy of our joint calibration and aberration correction framework in enhancing depth estimation accuracy (improving mean precision from 0.83 to 0.24 mm—a ${\sim }3.5\times$ enhancement; Fig. 3e). Tests on another weak-texture target (Fig. S14) with continuously varying depth (a 3D-printed flower, at an observation distance of 0.1 m) further confirm that MDS consistently delivers high-accuracy depth measurements (0.61 mm at 0.1 m) in this more challenging scenario. These results demonstrate the substantial promise of the MDS for industrial inspection scenarios, including bumps/bonding wire measurement, measurement of difficult-to-access pins and quality inspection of small parts.

**Figure 3.**
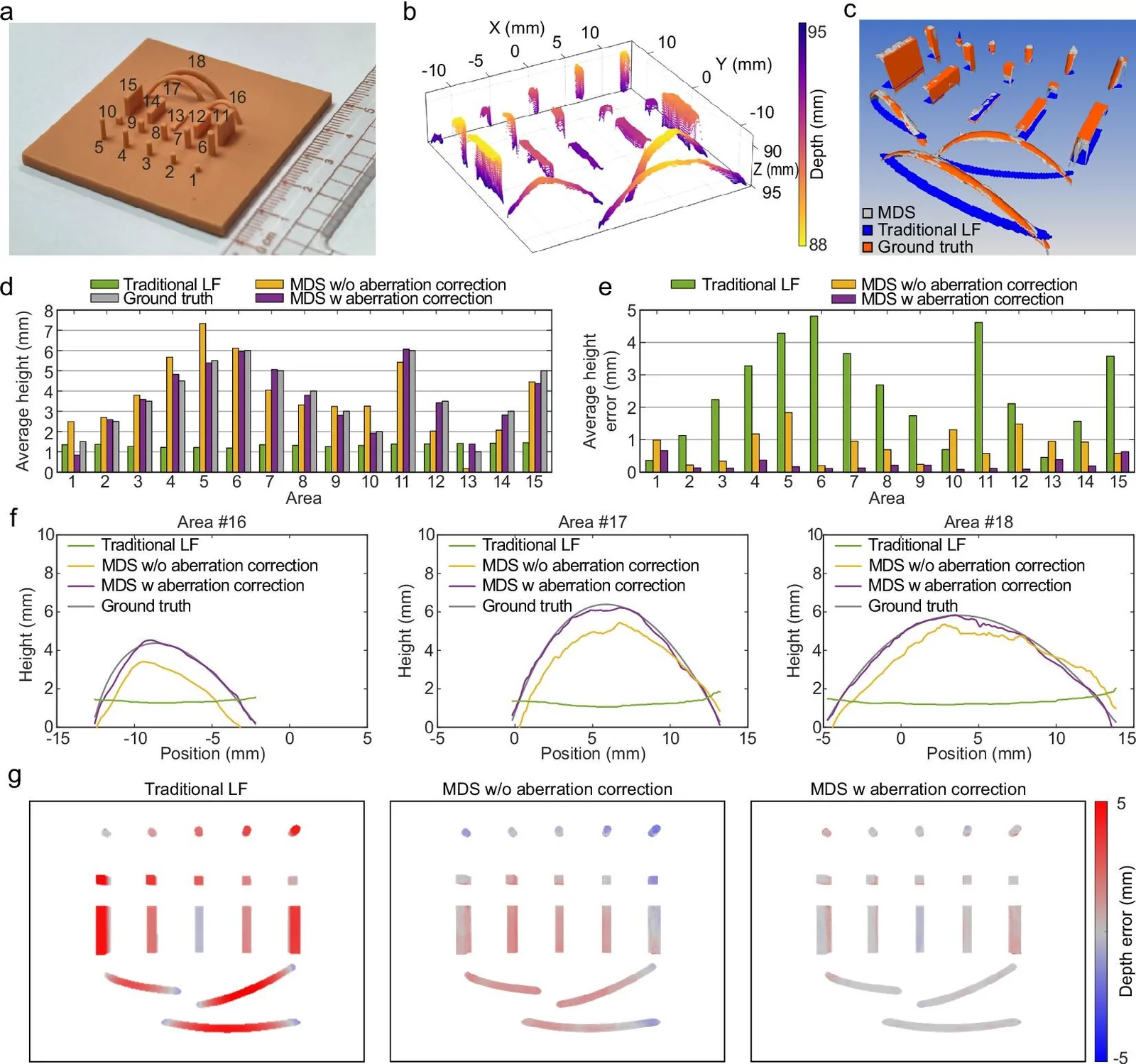
Close-range fine-grained depth perception. (a) High-precision 3D-printed target for close-range depth assessment, featuring microstructures (diameter/thickness: 1.2 mm) including pillars, thin plates and curved tubes of varying heights, simulating miniature industrial components. (b) Reconstructed 3D point cloud of microstructures obtained via MDS. (c) Comparative visualization of 3D mesh reconstructions for microstructures: ground truth versus MDS and conventional light-field imaging. (d) Comparative assessment of mean height estimation accuracy for thin pillars and plates across methods: conventional light-field imaging, MDS without aberration correction and aberration-corrected MDS. (e) Mean height estimation error comparison for thin pillars and plates across evaluated methods. (f) Reconstructed ridge lines of curved tubes versus *X*-axis coordinate position, comparing conventional light-field imaging, uncorrected MDS and aberration-corrected MDS. (g) Depth estimation error distributions across microstructural features for all tested methodologies.

By integrating the MDS with a reflective telescope assembly (Section S7), we observed targets at 300- and 500-m distances (Fig. 4 a and b). Leveraging the system’s digital adaptive optics capability [33] and the turbulence-induced scanning [38] algorithm with multi-frame fusion processing (see Section S7), we achieve turbulence-robust depth estimation, maintaining stable measurement accuracy even at a 500-m distance (Fig. 4c). Depth variation analysis across test scenarios shows that our aberration-corrected framework produces measurements that better align with object profiles compared to conventional light-field systems and uncorrected implementations (Fig. 4d), further verifying its operational reliability in practical environments. For observation ranges exceeding 300 m, high-end LiDAR systems typically remain the standard for reliable depth measurement. While LiDAR achieves centimetre-level ranging accuracy, it produces sparse point clouds ($0.01^\circ$ angular resolution) and fails to reliably resolve thin structural elements (Fig. 4b, cylindrical pillars), low-reflectivity objects (Fig. 4b, tree branches) and inclined surfaces (Fig. 4b, stadium dome) due to insufficient laser return signals. In contrast, the MDS enables fine-grained depth estimation for thin structures (Scenario 1, Fig. 4c), low-reflectivity targets (Scenario 2, Fig. 4c) and large-scale inclined surfaces (Scenario 3, Fig. 4c), with reconstruction accuracy significantly surpassing conventional light-field imaging (Fig. 4c and d). We further quantitatively assessed the long-range depth estimation accuracy of MDS on a building surface featuring a raised letter ‘a’ with LiDAR ground-truth measurements (Fig. 4e and f). Experimental results demonstrate that MDS generates denser 3D point clouds (Fig. 4g) compared with commercial LiDAR systems, enabling superior reconstruction of surface features such as the raised letter. Moreover, MDS achieves a depth estimation precision of 0.7 m at a 235-m distance (Fig. 4h), corresponding to a relative error of 0.3%.

**Figure 4.**
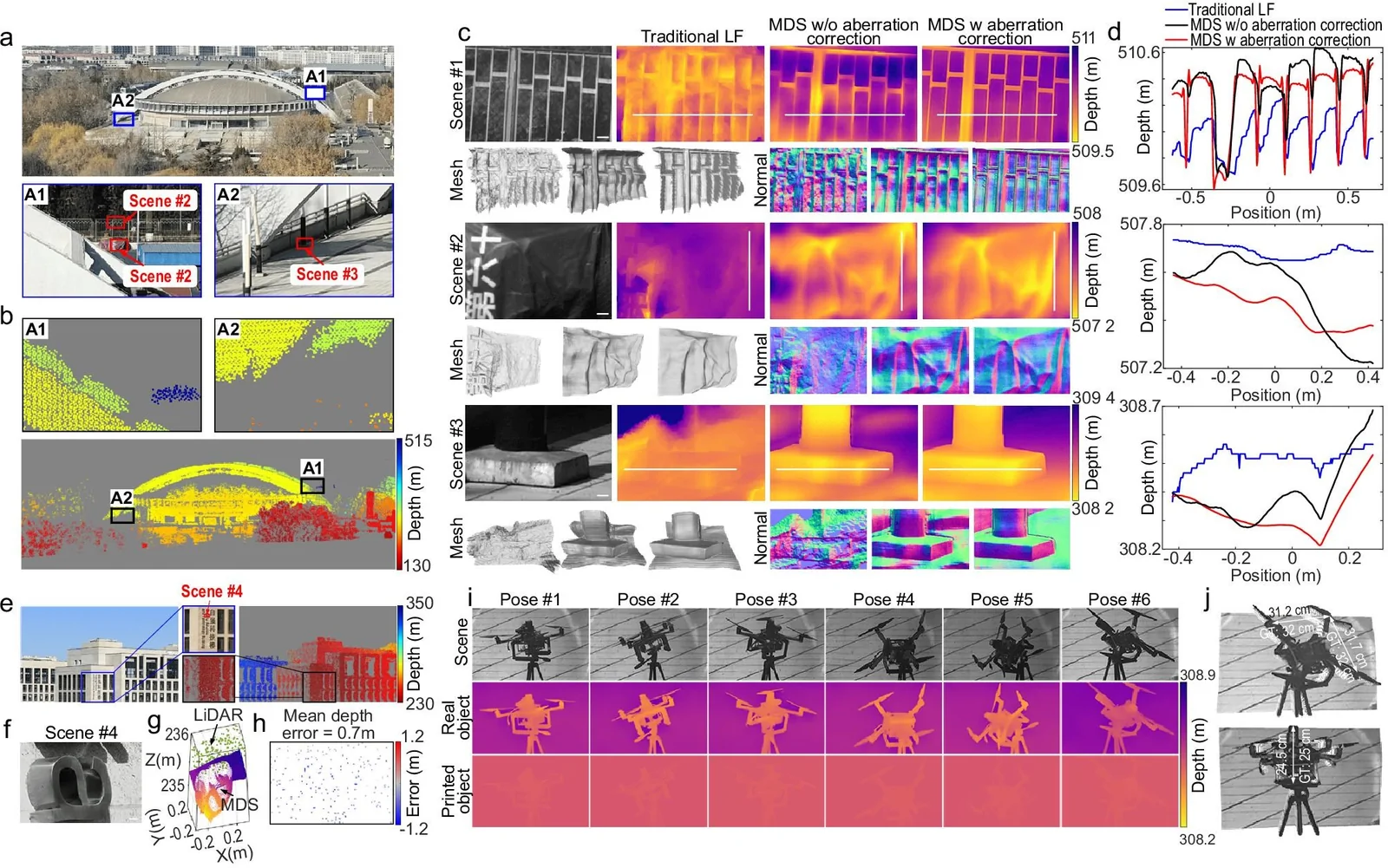
Long-range fine-grained depth perception. (a) The image of a long-distance scene (part of Tsinghua University campus) captured with a smartphone. (b) Sparse point clouds correspond to the scene captured with a long range LiDAR. (c) Depth estimation results and corresponding 3D meshes with surface normal maps for three long-range scenes (indicated by red rectangles in (a)), comparing traditional light-field imaging with the proposed MDS both with and without aberration correction. (d) Depth profiles of the white lines marked in (c). (e) Another long-distance scene (a building of Tsinghua University) and the corresponding LiDAR point clouds. (f) Test scene for long-range depth estimation accuracy evaluation. (g) Three-dimensional point cloud reconstruction of test scene #4 using MDS, with corresponding LiDAR-captured sparse point clouds. (h) The depth estimation error distributions of our MDS method, using LiDAR measurements as ground truth, demonstrate that MDS achieves a depth estimation precision of 0.7 m at a distance of 235 m (0.3% relative accuracy). (i) Long-range drone depth estimation tests. We employed MDS for depth estimation of drones under various poses. Additionally, to simulate a printed planar drone model, the center view was warped to adjacent subviews with zero disparity and additive white Gaussian noise emulating real-world printed conditions. Corresponding depth estimation results consistently aligned within a common plane, demonstrating MDS’s capability to reliably distinguish authentic drones from printed replicas. (j) Dimensional measurements conducted on the drone point cloud generated via MDS estimation. At a standoff distance of 300 m, the dimensional measurement error remained within 3%. Scale bars: 0.11 m ((c), scene #1), 0.11 m ((c), scene #2), 0.065 m ((c), scene #3), 0.05 m ((f), scene #4).

The long-range fine-grained depth perception capability of MDS endows it with potential application value in the remote positioning and metrology of drones. We applied MDS to perform depth estimation for a static drone at 300 m under various poses (Fig. 4i). The results demonstrate that MDS not only achieves accurate distance localization but also captures fine structural depth details of the drone. To simulate a spoofing scenario involving a printed planar drone, we warped the center-view image to other subviews via zero-disparity projection, with additive white Gaussian noise applied to each subview image to emulate realistic imaging conditions. The resulting depth estimates consistently lay near the same plane, indicating that MDS can reliably distinguish real drones from printed decoys. Furthermore, size measurements performed on the drone’s dense point cloud generated by MDS show that, for a small drone measuring $32\times 32\times 25$ cm$^3$ at 300 m, the measurement error remains within 3% (Fig. 4j). These experiments suggest that MDS holds considerable potential for low-altitude drone reconnaissance, positioning, and trajectory tracking and prediction.

The results in Figs 3 and 4 show that the complete MDS system can directly achieve cross-scale depth estimation by simply pairing it with different lenses, without any need to modify the algorithm or model. Therefore, although cross-scale depth imaging inherently requires lenses of different apertures and focal lengths, MDS, as a unified framework, achieves a substantial improvement in both the accuracy and throughput of cross-scale depth estimation.

### Temporally consistent video ranging

Depth perception of dynamic scenes holds critical importance for applications including autonomous driving, robotics and AR/VR systems. Addressing high-throughput dynamic depth sensing necessitates simultaneous achievement of real-time reconstruction and temporal coherence in inter-frame depth estimation. To resolve this dual requirement, we developed a unified computational architecture integrating virtual-scanning modalities with video depth stabilization paradigms, achieving stabilized, high-fidelity video depth perception at 3-megapixel resolution at up to the camera frame rate.

The mechanical two-axis galvo scanning inherently operates in division-of-time mode, rendering it fundamentally unsuitable for dynamic scene depth estimation. To address this limitation, we leverage the state-of-the-art virtual-scanning technique [36] to replace mechanical scanning, enabling high-throughput subaperture image reconstruction through snapshot light-field acquisition and computational processing (Fig. 5a). By training the virtual-scanning network on macroscale datasets (Section S8), we bypass mechanical scanning limitations and motion artifacts, achieving scanning light-field equivalent multi-view imaging at camera frame rates, thus enabling video-rate high-throughput depth estimation.

**Figure 5.**
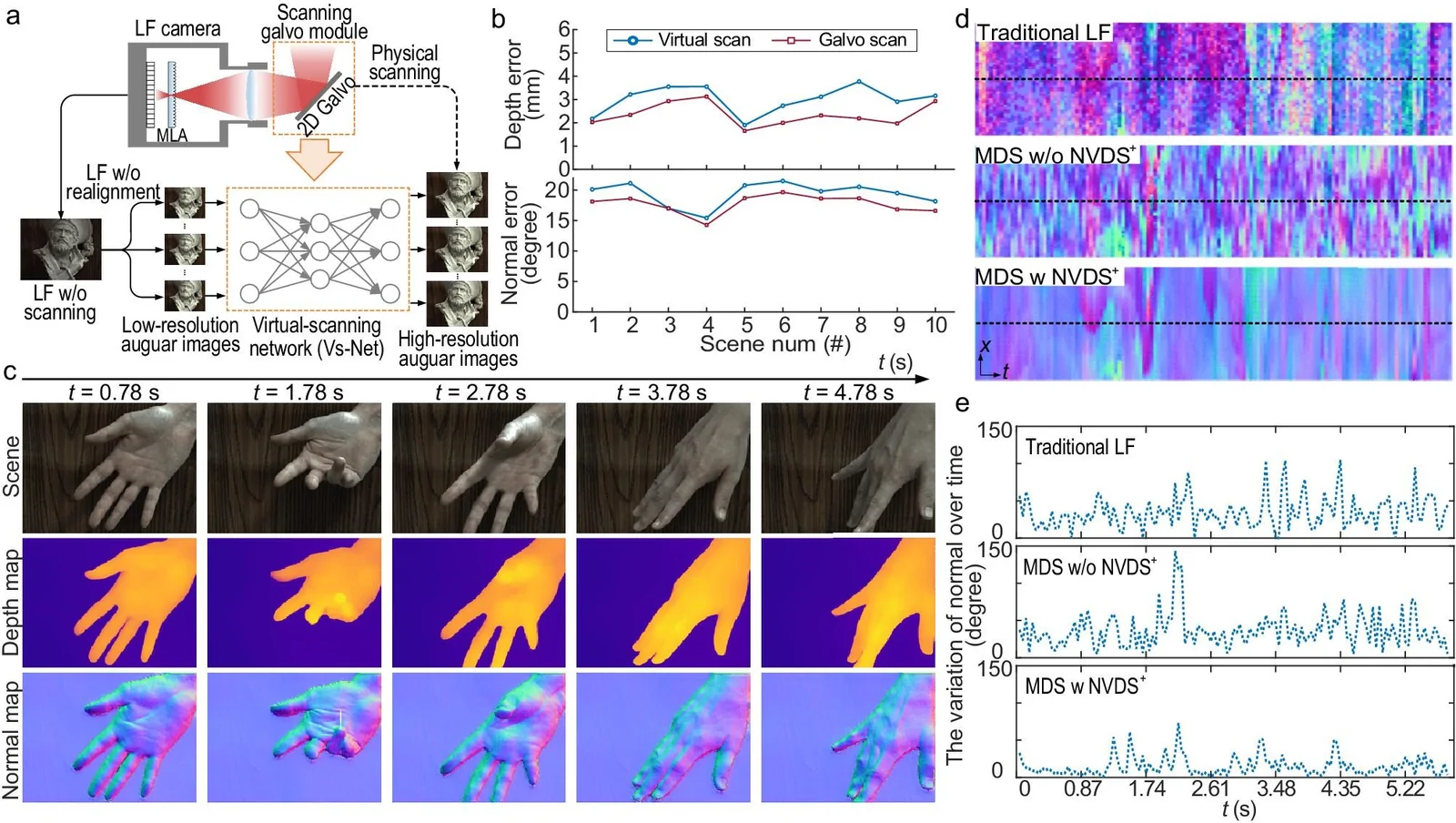
Virtual scanning enables temporally consistent video ranging. (a) Schematic of the virtual-scanning approach, which replaces galvo scanning to reconstruct high-throughput subaperture images. (b) Comparison of depth and normal estimation performance between galvo-scanning light-field and virtual-scanning light-field imaging. (c) Temporal depth estimation of a moving hand; depth and corresponding normal maps are provided. (d) Kymograph space-time (*x*-*t*) plot corresponding to the white lines marked in (c) via traditional light-field imaging, MDS and MDS with refined NVDS$^+$, captured over approximately 6 s. (e) The variation of normal over time along the dashed line marked in (d).

Performance evaluation demonstrates that metric depth estimation using virtually scanned subaperture images achieves comparable accuracy to mechanical scanning in both depth estimation (mean error *< *3.3 mm) and surface normal reconstruction (mean angular error ${< }18.9^\circ$), with negligible precision degradation (Fig. 5b). The performance degradation (Fig. 5b and Fig. S16) caused by virtual scanning is very limited relative to mechanical scanning, yet the gain it offers is high-throughput depth sensing of dynamic scenes, which cannot be achieved by mechanical scanning alone. While our frame-wise depth estimation algorithm inherently exhibits temporal discontinuity due to inter-frame variance, we employ a fine-tuned Neural Video Depth Stabilizer (NVDS$^+$) [39] to enhance inter-frame continuity through post-processing (Section S9). This hybrid framework of virtual scanning and NVDS$^+$ enables continuous high-throughput absolute depth video reconstruction from low-resolution light-field video streams at native camera frame rates.

For dynamic object testing, we captured light-field video of a moving hand and reconstructed depth frames using virtual scanning, with final stabilization through NVDS$^+$ (Fig. 5c). The system successfully resolves continuous high-throughput depth maps and surface normals without motion artifacts, reconstructing fine anatomical details such as palm lines and vascular protrusions (Fig. 5c). Quantitative analysis confirms our framework’s enhanced temporal stability, showing clear reduction in depth jitter compared to conventional light-field methods and unoptimized implementations (Fig. 5d and e). Additional dynamic reconstruction results are provided in Videos S1–S3.

## CONCLUSION

This study introduces the MDS, a paradigm shift in depth perception by harmonizing aberration-robust disparity-depth calibration and depth-vision-foundation-model-empowered fine-grained 3D ranging in a scanning light-field imaging framework. Traditional approaches—whether active (LiDAR, structured light) or passive (stereo vision, monocular networks)—have been constrained by the trade-off between precision and throughput. The MDS resolves these limitations through three synergistic advances. (1) A scanning light-field meta-imaging paradigm enables high-precision metric depth estimation via high-throughput disparity regression. Both physical scanning and computational virtual scanning enhance spatial resolution by increasing subaperture sampling density, thereby improving depth estimation accuracy without losing details caused by occlusions. (2) With this framework, we achieved monocular high-precision, fine-grained metric depth estimation across scales from 0.1 to 500 m in a fully passive manner. For dynamic scenes, our framework provides high-throughput, temporally continuous metric depth estimation at up to the camera frame rate. (3) We proposed a system-level aberration-robust disparity-depth calibration method to address both optical imperfections and geometric inconsistencies. Integrating vision foundation model priors with physical light-field constraints produces detail-preserving absolute depth maps, circumventing scale ambiguity in pure learning-based approaches and detail insufficiency in conventional stereo matching.

The demonstrated 0.18% relative depth accuracy at the 1.5-m distance rivals high-end structured-light systems while maintaining five-fold higher resolution, addressing a critical gap in applications requiring high depth precision and high spatial resolution. The MDS enables submillimeter depth estimation accuracy (0.24-mm mean error) for microstructures at a 0.1-m working distance, achieving a 10*×* precision enhancement over conventional light-field imaging and revealing transformative potential for critical industrial inspection scenarios, including bonding wire metrology, confined-pin measurement and micro-component quality assessment. The MDS’s long-range performance (0.7-m depth precision at a 235-m distance) particularly highlights its potential in autonomous systems and remote sensing, where LiDAR’s sparse sampling and texture-dependent passive methods fail. For dynamic scenes, virtual scanning enables real-time depth estimation without mechanical trade-offs, achieving temporally consistent high-throughput absolute depth estimation. However, as a purely passive, triangulation-based depth-sensing method, MDS inherently relies on scene texture compared with active techniques such as LiDAR, which is a limitation shared by MDS and existing passive depth-sensing technologies alike. Our algorithm still struggles with extreme textureless scenes, such as a field of view consisting entirely of a textureless white wall or a blank sheet of paper. More advanced computational imaging techniques may address this issue in the future, and we will also explore solutions in our future work.

By bridging the chasm between metrology-grade accuracy and photographic resolution, the MDS redefines the possibilities for 3D perception. Applications span from turbulence-robust long-range 3D imaging to near-field depth measurement, underscoring its transformative potential across scales previously deemed mutually exclusive in computational imaging.

## Supplementary Material

nwag524_Supplemental_Files

## Data Availability

The codes for the whole pipeline of the MDS and the relevant data are available at https://github.com/LeeNeo110/MDS.

## References

[1] Van Brummelen J, O’Brien M, Gruyer D et al. Autonomous vehicle perception: the technology of today and tomorrow. Transp Res Pt C-Emerg Technol 2018; 89: 384–406. 10.1016/j.trc.2018.02.012

[2] Lelek M, Gyparaki MT, Beliu G et al. Single-molecule localization microscopy. Nat Rev Method Prim 2021; 1: 39. 10.1038/s43586-021-00038-x PMC9160414 35663461

[3] Rogers C, Piggott AY, Thomson DJ et al. A universal 3D imaging sensor on a silicon photonics platform. Nature 2021; 590: 256–61. 10.1038/s41586-021-03259-y 33568821

[4] Paredes AL, Song Q, Conde MH. Performance evaluation of state-of-the-art high-resolution time-of-flight cameras. IEEE Sens J 2023; 23: 13711–27. 10.1109/JSEN.2023.3273165

[5] Laga H, Jospin LV, Boussaid F et al. A survey on deep learning techniques for stereo-based depth estimation. IEEE Trans Pattern Anal Mach Intell 2020; 44: 1738–64. 10.1109/TPAMI.2020.3032602 33079659

[6] Yang L, Kang B, Huang Z et al. Depth anything: unleashing the power of large-scale unlabeled data. In: Proceedings of the IEEE/CVF Conference on Computer Vision and Pattern Recognition. Piscataway, NJ: IEEE Press, 2024, 10371–81.

[7] Floreano D, Wood RJ. Science, technology and the future of small autonomous drones. Nature 2015; 521: 460–6. 10.1038/nature14542 26017445

[8] Bao F, Wang X, Sureshbabu SH et al. Heat-assisted detection and ranging. Nature 2023; 619: 743–8. 10.1038/s41586-023-06174-6 37495879

[9] Gwosch KC, Pape JK, Balzarotti F et al. MINFLUX nanoscopy delivers 3D multicolor nanometer resolution in cells. Nat Methods 2020; 17: 217–24. 10.1038/s41592-019-0688-0 31932776

[10] Karunratanakul K, Yang J, Zhang Y et al. Grasping field: Learning implicit representations for human grasps. In: 2020 International Conference on 3D Vision (3DV). Los Alamitos, CA: IEEE Computer Society, 2020, 333–44. 10.1109/3DV50981.2020.00043

[11] Almalioglu Y, Turan M, Trigoni N et al. Deep learning-based robust positioning for all-weather autonomous driving. Nat Mach Intell 2022; 4: 749–60. 10.1038/s42256-022-00520-5 PMC10543073 37790900

[12] de Conto T, Armston J, Dubayah R. Characterizing the structural complexity of the Earth’s forests with spaceborne LiDAR. Nat Commun 2024; 15: 8116. 10.1038/s41467-024-52468-2 PMC11405527 39284819

[13] Dorrah AH, Bordoloi P, de Angelis VS et al. Light sheets for continuous-depth holography and three-dimensional volumetric displays. Nat Photon 2023; 17: 427–34. 10.1038/s41566-023-01188-y

[14] He B, Zhang Y, Zhao L et al. Robotic-OCT guided inspection and microsurgery of monolithic storage devices. Nat Commun 2023; 14: 5701. 10.1038/s41467-023-41498-x PMC10502073 37709753

[15] Ibanez-Guzman J, Li Y. LiDAR and cameras in autonomous driving. Nat Rev Electr Eng 2025; 2: 515–6. 10.1038/s44287-025-00176-4

[16] Chen R, Shu H, Shen B et al. Breaking the temporal and frequency congestion of LiDAR by parallel chaos. Nat Photon 2023; 17: 306–14. 10.1038/s41566-023-01158-4

[17] Roriz R, Cabral J, Gomes T. Automotive LiDAR technology: a survey. IEEE Trans Intell Transp Syst 2021; 23: 6282–97. 10.1109/TITS.2021.3086804

[18] Jeong D, Jang H, Jung MU et al. Spatio-spectral 4D coherent ranging using a flutter-wavelength-swept laser. Nat Commun 2024; 15: 1110. 10.1038/s41467-024-45297-w PMC10847489 38321004

[19] Zhang X, Kwon K, Henriksson J et al. A large-scale microelectromechanical-systems-based silicon photonics LiDAR. Nature 2022; 603: 253–8. 10.1038/s41586-022-04415-8 PMC8907073 35264759

[20] Giancola S, Valenti M, Sala R. A Survey on 3D Cameras: Metrological Comparison of Time-of-Flight, Structured-Light and Active Stereoscopy Technologies, Cham: Springer, 2018. 10.1007/978-3-319-91761-0

[21] Kim G, Kim Y, Yun J et al. Metasurface-driven full-space structured light for three-dimensional imaging. Nat Commun 2022; 13: 5920. 10.1038/s41467-022-32117-2 PMC9550774 36216802

[22] Yi L, Hou B, Zhao H et al. X-ray-to-visible light-field detection through pixelated colour conversion. Nature 2023; 618: 281–6. 10.1038/s41586-023-05978-w PMC10247359 37165192

[23] Godard C, Mac Aodha O, Firman M et al. Digging into self-supervised monocular depth estimation. In: Proceedings of the IEEE/CVF International Conference on Computer Vision. Piscataway, NJ: IEEE Press, 2019, 3827–37.

[24] Yang L, Kang B, Huang Z et al. Depth anything v2. In: Proceedings of the 38th International Conference on Neural Information Processing Systems. Red Hook, NY: Curran Associates, 2024, 21875–911.

[25] Tang H, Cohen S, Price B et al. Depth from defocus in the wild. In: Proceedings of the IEEE Conference on Computer Vision and Pattern Recognition. Piscataway, NJ: IEEE Press, 2017, 4773–81.

[26] Opatovski N, Xiao D, Harari G et al. Monocular kilometer-scale passive ranging by point-spread function engineering. Opt Express 2022; 30: 37925–37. 10.1364/OE.472150 36258371

[27] Shen Z, Zhao F, Jin C et al. Monocular metasurface camera for passive single-shot 4D imaging. Nat Commun 2023; 14: 1035. 10.1038/s41467-023-36812-6 PMC9950364 36823191

[28] Ng R. Digital light field photography. Ph.D. Thesis, Stanford University, 2005.

[29] Tsai YJ, Liu YL, Ouhyoung M et al. Attention-based view selection networks for light-field disparity estimation. In: Proceedings of the AAAI Conference on Artificial Intelligence. Washington, DC: AAAI Press, 2020, 12095–103. 10.1609/aaai.v34i07.6888

[30] Williem , Park IK, Lee KM. Robust light field depth estimation using occlusion-noise aware data costs. IEEE Trans Pattern Anal Mach Intell 2018; 40: 2484–97. 10.1109/TPAMI.2017.2746858 28866482

[31] Li P, Zhao J, Wu J et al. OPAL: occlusion pattern aware loss for unsupervised light field disparity estimation. IEEE Trans Pattern Anal Mach Intell 2023; 46: 681–94. 10.1109/TPAMI.2023.3296600 37463080

[32] Wilburn B, Joshi N, Vaish V et al. High performance imaging using large camera arrays. ACM Trans Graph 2005; 24: 765–76. 10.1145/1073204.1073259

[33] Wu J, Guo Y, Deng C et al. An integrated imaging sensor for aberration-corrected 3D photography. Nature 2022; 612: 62–71. 10.1038/s41586-022-05306-8 PMC9712118 36261533

[34] Cao Z, Li N, Zhu L et al. Aberration-robust monocular passive depth sensing using a meta-imaging camera. Light-Sci Appl 2024; 13: 236. 10.1038/s41377-024-01609-9 PMC11377717 39237492

[35] Liao W, Zhu Y, Wang X et al. LightM-UNet: Mamba assists in lightweight UNet for medical image segmentation [preprint]. arXiv: 2403.05246.

[36] Lu Z, Liu Y, Jin M et al. Virtual-scanning light-field microscopy for robust snapshot high-resolution volumetric imaging. Nat Methods 2023; 20: 735–46. 10.1038/s41592-023-01839-6 PMC10172145 37024654

[37] Chen J, Hou J, Ni Y et al. Accurate light field depth estimation with superpixel regularization over partially occluded regions. IEEE Trans Image Process 2018; 27: 4889–900. 10.1109/TIP.2018.2839524 29969399

[38] Guo Y, Hao Y, Wan S et al. Direct observation of atmospheric turbulence with a video-rate wide-field wavefront sensor. Nat Photon 2024; 18: 935–43. 10.1038/s41566-024-01466-3

[39] Wang Y, Shi M, Li J et al. NVDS+: towards efficient and versatile neural stabilizer for video depth estimation. IEEE Trans Pattern Anal Mach Intell 2024; 47: 583–600. 10.1109/TPAMI.2024.3476387 39378259

