# Supplementary material for: High-throughput and accurate cross-scale 3D sensing with a meta-depth sensor
Source: Natl Sci Rev. 2026 Aug 21;13(20):nwag524. doi: 10.1093/nsr/nwag524 (PMC13702026; doi:10.1093/nsr/nwag524)
Supplement: Supplementary file 1 [file nwag524_supplemental_files.zip › MDS_Supplementary.pdf]

Supplementary information

High-Throughput and Accurate Cross-Scale 3D Sensing with a Meta-Depth Sensor

Tao Yu[1†], Ning Li[2†], Qingqian Lang[1], Zhexuan Cao[3,4], Yuhan Hao[3,4], Laiyu Zhu[3,4], Jiamin Wu[1,3,4*] & Qionghai Dai[1,3,4*]

[1]*Beijing National Research Center for Information Science and Technology, Tsinghua University, Beijing 100084, China*

[2]*School of Electronic and Information Engineering, Zhengzhou University, Zhengzhou 450001, China*

[3]*Department of Automation, Tsinghua University, Beijing 100084, China*

[4]*Institute for Brain and Cognitive Sciences, Tsinghua University, Beijing 100084*

[†]*These authors contributed equally to this work*

[*]Correspondence: wujiamin@tsinghua.edu.cn (J. W.), qhdai@tsinghua.edu.cn (Q. D.)

**Supplementary Section S1**	System and experimental setup
**Supplementary Section S2**	Joint aberration-disparity calibration
**Supplementary Section S3**	Network architecture
**Supplementary Section S4**	Loss function
**Supplementary Section S5**	Training data and setup
**Supplementary Section S6**	Close-range depth estimation
**Supplementary Section S7**	Long-range depth estimation
**Supplementary Section S8**	Virtual scanning
**Supplementary Section S9**	Video depth temporal continuity enhancement
**Supplementary Figure S1**	Comparison of light-field image reconstruction results before and after correction with the proposed compensation algorithm.
**Supplementary Figure S2**	Joint aberration-disparity calibration algorithm.
**Supplementary Figure S3**	Overview of depth vision Foundation model guided Mamba network (FMNet) architecture.
**Supplementary Figure S4**	Disparity estimation results on weak-texture scenes.
**Supplementary Figure S5**	Depth measurement results on objects with varying albedo.
**Supplementary Figure S6**	The effectiveness of the depth vision foundation model.
**Supplementary Figure S7**	Evaluation of traditional light-field depth estimation algorithms on scanning light-field data.
**Supplementary Figure S8**	Training strategy utilizing depth foundation model predictions to constrain the proposed FMNet.
**Supplementary Figure S9**	Data used for model training and testing.
**Supplementary Figure S10**	Influence of using synthetic data for training.
**Supplementary Figure S11**	Weighted patch fusion for high-throughput depth reconstruction.
**Supplementary Figure S12**	Generalization test of the proposed FMNet on urban autonomous driving scenes.

**Supplementary Figure S13**	Experimental setup for close-range depth estimation.
**Supplementary Figure S14**	Depth estimation experiment on a continuously varying depth target (a 3D-printed flower) at an observation distance of 0.1 m.
**Supplementary Figure S15**	Long range light-field imaging system.
**Supplementary Figure S16**	Comparison of light-field image reconstruction quality and depth estimation results between virtual scanning and galvo scanning.
**Supplementary Figure S17**	Fine-tuning scheme of $NVDS^{+}$.
**Supplementary Video 1**	Video depth estimation of rotating sculpture.
**Supplementary Video 2**	Video depth estimation of moving hand.
**Supplementary Video 3**	Video depth estimation of shaking paper.

Section S1: System and experimental setup

The MDS integrates a Lingyun Optoelectronics LBAS-U3120E-29C/M industrial camera with microlens array (MLA). The camera employs a Sony IMX226 color sensor with 4000×3036 resolution (1.85 μm pixel pitch) operating at 29 fps maximum frame rate. The MLA contains 14.8×14.8μm microlenses (F/2, 29.6 μm focal length), each corresponding to an 8×8 pixel region on the sensor, providing 64 discrete viewing perspectives. The main lens maintains F/2 configuration with 50 mm focal length - this optical design maximizes system aperture for enhanced depth estimation accuracy through extended equivalent baseline.

Each microlens incorporates a chromium-coated circular mask pattern to block the light passing through the surrounding corners of the square footprint of each microlens. Scanning functionality is implemented via an external two-axis galvo mirror (Coremorrow P33.T8SF piezoelectric tilt stage) positioned at 45° to the optical axis. A custom control board synchronizes mirror scanning with image acquisition through hardware-triggered synchronization. Despite these measures, minor deviations of the scanning trajectory from the ideal rectangular grid are inevitable. We found that such deviations cause sawtooth-like artifacts in the reconstructed multi-view images, with the artifacts increasing approximately linearly from the center to the edge of the field of view. To address this problem, we designed a post-processing algorithm that compensates for these errors during pixel realignment, thereby improving the quality of the reconstructed multiview images. Figure S1 demonstrates the effectiveness of the proposed compensation method.

For all experiments except long-range testing, we employ 4×4 scanning patterns with image preprocessing (including rectification and pixel realignment[33]) to generate multi-view RGB light-field images at 1924×1444 resolution. Conventional light-field imaging comparisons use the same hardware configuration without scanning,

capturing 481×361 resolution images from central scanning positions to ensure consistent observation geometry.

Section S2: Joint aberration-disparity calibration

In light-field imaging, the theoretical precision limit for metrin depth estimation through disparity estimation can be expressed as:

$$\Delta z = \frac{z^2}{Bf} \cdot \Delta D \tag{1}$$

where z denotes target distance, B the equivalent baseline, f the focal length, and $\Delta D = \alpha p$ represents disparity estimation accuracy with α being algorithmic precision and p pixel size. This formulation indicates that under fixed system parameters, enhancing depth estimation precision requires improving either algorithmic disparity accuracy or effective pixel resolution through increased scanning density. While higher scanning counts reduce effective pixel size, two-dimensional scanning induces quadratic growth in total scan numbers, creating impractical data volumes without overcoming optical diffraction limits. Our system employs 4×4 scanning to balance acquisition efficiency and depth resolution.

Absolute depth estimation via scanning light-field imaging requires established inverse proportionality between disparity and depth parameters along with strict bijective correspondence between disparity and depth values. Although ideal systems permit direct derivation of disparity-depth relationship parameters from optical specifications, manufacturing imperfections introduce aberrations that prevent direct parameter calculation and induce disparity-depth inconsistencies.

To address these challenges, we propose an integrated framework that combines depth-disparity calibration with aberration correction for scanning light-field imaging systems. This approach utilizes high-precision active depth imaging devices (such as structured-light cameras or ToF cameras) to acquire depth information while

simultaneously capturing corresponding light-field data through the scanning light-field imaging system. Through iterative optimization, we achieve coordinated calibration of both distortion correction maps for each viewing perspective and the inverse relationship parameters between disparity and depth, thereby improving the consistency between measured disparities and true depths in the scanning light-field system. For high-precision depth acquisition, we employed a Photoneo PhoXi 3D Scanner L structured-light camera with a measurement range of 870-2150 mm, field of view of 1082 × 722 mm, and depth measurement accuracy better than 1 mm. The combined acquisition system of the scanning light-field imaging system and structured-light camera is shown in Fig. S2a.

The calibration procedure (Fig. S2a) begins with data collection, which primarily includes scanning light-field data and corresponding structured-light depth measurements of checkerboard calibration targets at various distances, as well as similar paired data for textured pattern boards. For checkerboard targets, the distribution should comprehensively cover the three-dimensional measurement space while avoiding excessively large patterns that would result in sparse 3D distributions of corner points and compromise calibration accuracy. The specific patterns on texture boards are not constrained, provided they contain easily distinguishable features.

For checkerboard data, we detect corresponding corner points across different sub-aperture views and calculate the disparity at each corner using their image coordinates. Since the structured-light camera simultaneously records grayscale texture information, corner detection can also extract corresponding depth measurements. It should be noted that the acquired structured-light depth information follows the world coordinate system of the structured-light camera, requiring transformation into the scanning light-field camera's coordinate system through extrinsic parameters to obtain ground-truth depth in the scanning light-field

coordinate frame. This necessitates prior calibration of the scanning light-field camera's intrinsic and extrinsic parameters, which can be achieved through standard calibration algorithms and will not be elaborated here. This process yields a series of depth-disparity pairs that form the foundation for subsequent joint calibration and correction in the meta-imaging system.

Our proposed joint aberration-disparity correction framework comprises two interconnected components: depth-disparity parameter calibration and multi-view aberration correction. The calibration component establishes the inverse relationship between system disparity and depth measurements, while the aberration correction component minimizes disparity-depth inconsistencies through aberration compensation of multi-view measurements. These components are co-optimized through an iterative process to achieve system-level absolute depth estimation optimization.

The depth-disparity relationship is modeled as:

$$d = \frac{a}{z + b} + c \tag{2}$$

where d represents disparity, z denotes depth, and a, b, c are calibration parameters. These parameters are iteratively updated using depth-disparity pairs obtained from checkerboard corner detection across multiple calibration planes.

For aberration correction, we first optimize low-resolution correction maps (20×15 pixels) for all non-central view images. These maps are then upsampled to each view's native resolution (1920×1440 pixels) to perform non-rigid geometric transformations that compensate for optical aberrations.

To optimize both the depth-disparity inverse relationship parameters and the low-resolution aberration correction maps for each sub-view images, we designed two loss functions to constrain and optimize the learning process. The first component is

the fitting loss, which constrains fitting error obtained by disparity and depth data pairs from checkerboard patterns. By correcting aberrations across different sub-view images using the correction maps, the fitting error is gradually reduced. The fitting constraint loss function is expressed as:

$$\mathcal{L}_{fit} = \frac{1}{N}\sum_{i=1}^{N} \left\| d_i - \widehat{d}_i \right\|_1 \tag{3}$$

where d_i represents the disparity calculated at the current corner using the ground truth depth and fitted parameters, $\widehat{d}_i$ denotes the disparity computed from corner detection on aberration-corrected sub-view images, and N is the total number of corners in the checkerboard data across different distances.

The second component is the sub-view image reprojection loss, which constrain the error between each reprojected sub-view image and center view image. Based on the fitted depth-disparity relationship, the corresponding disparity map is computed from the depth data. Then, the aberration corrected sub-view images of texture board are reprojected onto the center view images based on the disparity map to ensure the consistency. The sub-view image reprojection loss function is expressed as:

$$\mathcal{L}_{warp} = \frac{\sum_u \left\| \hat{I}_{u\to u_0}(x, D) - I_{u_0}(x) \right\|_1}{U \times M} \tag{4}$$

where $\hat{I}_{u\to u_0}(x, D)$ denotes the warped view-u image projected to center view u_0 using disparity D derived from texture board depth maps, $I_{u_0}(x)$ is the center reference view, U represents the number of warped views, and M indicates calibration dataset groups.

The composite loss function $\mathcal{L} = \mathcal{L}_{fit} + \mathcal{L}_{warp}$ drives simultaneous optimization of both depth-disparity parameters and aberration correction maps. Final implementation applies the upsampled correction maps to raw light-field data, followed by definitive

depth-disparity parameters fitting using calibrated checkerboard measurements. This joint calibration framework requires only one-time implementation per system configuration.

After the joint aberration-disparity correction process, the overall depth-disparity consistency of the MDS is significantly improved (Fig. S2b), the depth-disparity fitting error is reduced by a factor of five (Fig. S2c), and the depth estimation error is decreased by 34% (Fig. S2d). These improvements establish a robust foundation for high-throughput, high-precision metric depth estimation.

Section S3: Network architecture

Conventional light-field cameras typically feature odd-numbered sub-view images to establish a definitive central view. However, our imaging system employs an 8×8 grid of 64 sub-aperture images, necessitating selection of the (4,5) coordinate position as the center reference view. Given pronounced distortion in peripheral views, we utilize four central horizontal and vertical sub-views as inputs for disparity estimation (Fig. S3a).

The proposed depth vision-foundation-model-guided vision-Mamba disparity reconstruction network (FMNet) integrates five core modules: (1) Weight-shared feature extractor using Spatial Pyramid Pooling (SPP) [31,40] to capture hierarchical contextual features across sub-aperture views. (2) Matching cost volume construction through disparity-wise feature traversal. (3) Cost processing via Mamba-based state space models (SSMs) [35] . (4) Disparity regression module. (5) Refinement module incorporating Depth Anything v2 (DA-v2)[24].

Unlike conventional 3D convolution approaches [29], our architecture constructs 2D cost volumes by fusing disparity and feature channel dimensions. Grouped convolution compresses feature channels per disparity level before processing through

Mamba modules. Initial disparity estimates D_{raw} undergo refinement through DA-v2-guided optimization, yielding final outputs D_{final} via residual learning.

Modern SSMs like Mamba[41] demonstrate linear computational complexity relative to input size while establishing long-range dependencies. Building upon LightM-UNet [35], we implement Residual Vision Mamba (RVM) layers containing Vision State-Space (VSS) modules for spatial-contextual learning (Fig. S3b). The cost processor sequentially applies one RVM layer, two RVM residual blocks (each containing dual RVM layers), and a final RVM layer.

While depth vision foundation models like DA-v2[24] achieve detailed relative depth estimation from single images, their absolute depth accuracy remains limited. Our framework synergizes DA-v2's detail-preserving capability with light-field physical constraints through a disparity refinement module (Fig. S3c). This module first employs DA-v2 to predict the relative disparity D_{da} of the central viewpoint. Subsequently, D_{da} is normalized to the same scale range as the initial parallax regression result D_{raw} of the scanning light field, yielding D_{norm}. Next, D_{raw} and D_{norm} are concatenated and fed into a lightweight residual refinement network (RFN) [42] to learn the residual information, thereby obtaining the final disparity estimation result D_{final}.

This hybrid architecture combines Mamba's physical disparity learning with DA-v2's texture-agnostic depth prior, enabling high-precision absolute depth estimation with enhanced detail resolution and robustness in textureless regions. As shown in Fig. S4, with the assistance of DA, the proposed FMNet is able to estimate smooth and reasonable disparity even in weak-texture regions, significantly outperforming both traditional light-field disparity estimation algorithms and FMNet without DA.

A common problem with passive depth imaging methods is that changes of the surface albedo can get imprinted into the estimated depth map -- e.g. a flat piece of paper with printed text could produce a depth map with the letters embossed into the geometry. We further performed a depth reconstruction test on a static white paper with printed letters. Compared with MDS without the assistance of a vision foundation model, the vision-foundation-model-assisted MDS achieves significantly improved depth continuity and accuracy at regions of varying albedo on the smooth plane (Fig. S5). This benefit comes from our proposed architecture that fuses a vision foundation model with the physical prior of multi-view stereo geometry, which allows the algorithm to better capture global spatial semantics and to understand the continuously varying surface structure of the target on such albedo-varying smooth planes. These experimental results demonstrate that the proposed MDS technique can effectively suppress texture-copy artifacts induced by varying albedo on smooth surfaces.

Section S4: Loss function

To train the proposed disparity estimation network (FMNet) for high-precision and detailed metric depth estimation, we implement a joint constraint strategy combining disparity optimization with surface normal regularization.

The disparity constraint applies L_1 loss minimization to both initial regression results D_{raw} and final refined outputs D_{final} guided by DA-v2:

$$\mathcal{L}_{raw} = \frac{1}{T}\sum_{x\in X} M(x) \cdot \left\| D_{raw}(x) - D_{gt}(x) \right\|_1 \tag{5}$$

$$\mathcal{L}_{final} = \frac{1}{T}\sum_{x\in X} M(x) \cdot \left\| D_{final}(x) - D_{gt}(x) \right\|_1 \tag{6}$$

Here, $x \in X$ denotes pixel coordinates, $M(x)$ a binary mask indicating valid depth regions (1 for measurable areas, 0 otherwise), and T the total valid pixels. The mask addresses occlusion artifacts and sparse sampling limitations inherent to structured-light cameras.

For surface smoothness and detail preservation, we introduce cosine similarity loss[43] for normal vectors:

$$\mathcal{L}_{normal} = \frac{1}{H \times W} \sum_{i=1}^{H} \sum_{j=1}^{W} \left(1 - \langle N_{final}(i,j), N_{gt}(i,j) \rangle\right) \tag{7}$$

where N_{final} and N_{gt} represent unit-normalized estimated and ground truth surface normals, respectively, and N_{final} is derived from the final depth through calibrated depth-disparity conversion with D_{final}.

Direct linear combination of disparity and normal losses proves suboptimal due to error amplification from inaccurate early-stage disparity estimates. We therefore implement progressive constraint scheduling:

$$\mathcal{L}_{total} = \lambda \mathcal{L}_{raw} + (1 - \lambda)\mathcal{L}_{final} + \tau \mathcal{L}_{normal} \tag{8}$$

with $\lambda = 0.6$ balancing raw/final disparity losses. The normal loss weight τ follows phase-dependent scheduling:

$$\tau = \begin{cases} 0 & if\ 0 \leq t \leq \delta_1 \\ \dfrac{e_c - \delta_1 e_t}{(\delta_2 - \delta_1)e_t}\mu & if\ \delta_1 < t \leq \delta_2 \\ \mu & if\ \delta_2 < t \leq 1 \end{cases} \tag{9}$$

where $t = e_c/e_t$ indicates training progress ratio (e_c: current epoch, e_t: total epochs). Empirical parameters $\delta_1 = 0.66$, $\delta_2 = 0.83$, and $\mu = 0.025$ control normal constraint phasing. This progressive strategy initially prioritizes disparity learning before

gradually introducing normal regularization (Fig. S8c), enabling simultaneous optimization of absolute depth accuracy and surface detail recovery. The experimental results demonstrate that the surface normal estimation error is significantly reduced when using the algorithm guided by DA-v2 and constrained by surface normals (Fig. S8d).

While structured-light measurements provide absolute depth constraints, surface normal regularization requires dense geometric information to ensure smooth yet detailed depth estimation. Although depth vision foundation models like DA-v2 struggle with absolute depth accuracy, they demonstrate superior capability in relative depth estimation and detail preservation. We therefore develop a structured-light-guided depth deformation method that warps DA-v2 predictions to align with sparse structured-light measurements while retaining high-resolution details, enabling generation of dense surface normal ground truth (Fig. S8a).

The deformation process begins by normalizing DA-v2 depth predictions to match structured-light depth scales. Assuming continuous deformation within semantic categories, we employ the Segment Anything Model (SAM) [44] for semantic segmentation of center view images. For each semantic class, we optimize low-resolution deformation maps that are subsequently upsampled to full resolution. The warping process minimizes L_1 loss between adapted DA-v2 depth and structured-light measurements through iterative refinement:

$$\mathcal{L}_{deform} = \frac{1}{|\Omega|} \sum_{x \in \Omega} \left\| D'_{DA}(x) - D_{SL}(x) \right\|_1 \tag{10}$$

where D'_{DA} denotes the warped DA-v2 depth, D_{SL} the structured-light depth, and Ω the valid measurement region. This produces adapted DA-v2 depth maps that combine structured-light absolute accuracy with DA-v2's detail resolution (Fig. S8b). The final

surface normal ground truth N_{gt} is then computed from these deformed depth maps through differential geometry.

Section S5: Training data and setup

The training dataset combines experimentally captured scanning light-field images with structured-light depth measurements, from which 30 samples were randomly selected for training and 10 reserved for testing. To address limitations in scene diversity and disparity range within real-world data, we incorporate the synthetic HCI light-field dataset [45] containing 20 training samples. Each HCI sample provides 9×9 views (81 views in total) at 512×512 resolution with ground truth disparity and depth maps. Training and test data are shown in Fig. S9. To maintain consistency with our experimental system's view distribution, we select sub-view images around the center view following the same selected views pattern as real data acquisition (Fig. S10a). Additionally, we implement spaced view sampling from the HCI dataset to expand disparity coverage and random horizontal flipping for enhanced generalization. This hybrid training strategy significantly improves long-range depth estimation performance (Fig. S10b).

Network optimization employs the ADAM optimizer with initial learning rate 3×10^{-4}, halved every 2,000 epochs over 6,000 total epochs. Training uses randomly cropped 384×384 patches from input images. The PyTorch implementation runs on an NVIDIA RTX 3090 GPU with batch size 5. For inference, input dimensions are set to 448×448 to maintain consistency with training scale while minimizing computational overhead. Larger images undergo tiled processing with 448×448 patches (25% overlap between adjacent tiles) followed by weighted linear blending of overlapping regions for final prediction fusion (Fig. S11).

It is worth noting that mainstream deep-learning-based light-field depth estimation algorithms [29,31,46-48] also typically train on a limited number of scenes (often no more than 30), because these algorithms do not simply fit depth in a black-box manner from multi-view data. Instead, they construct cost volumes to learn cross-view feature matching relationships, which enables physically driven depth reconstruction. Our network follows a similar design philosophy, learning texture matching across views rather than overfitting to texture appearance, and thus generalizes across diverse textures. Moreover, the incorporation of a depth vision foundation model (DA-v2) trained with over 62 million images enhances scene generalization and performance in low-texture regions. The textures in our close-range (Fig. 3) and long-range (Fig. 4) test scenes differ substantially from those in the training data (Fig. S9), yet our method performs well on these scenes. This further validates that, by organically combining the universal spatial semantics from a vision foundation model with the physical geometry of multi-view light-field data, the proposed FMNet achieves robust generalization. To further verify this, we fine-tuned and evaluated FMNet on UrbanLF [49], a light-field dataset of an urban autonomous driving scenario. As shown in Fig. S12, the results yielded accurate depth estimates in both synthetic and real light-field images, further demonstrating the proposed method's cross-scene generalization capability.

Section S6: Close-range depth estimation

For close-range depth estimation, we employed the MDS depicted in Fig. 1b, substituting the primary lens with an f = 12 mm, F/2 lens to achieve an appropriate field of view at a 10 cm working distance (Fig. S13). The test target comprised a high-precision 3D-printed structure, fabricated with a dimensional tolerance ≤0.05 mm; consequently, the original 3D printing file served as the ground-truth depth reference. This target was printed entirely on a 50×50×3 mm substrate, with features distributed within a central 30×30 mm region. It included: five cylindrical pillars

(each 1.2 mm diameter, heights ranging from 1.5 mm to 5.5 mm), five square pillars (top surface: 1.2 mm side length, heights from 2 mm to 6 mm), five thin plates (1.2 mm thickness, heights from 1 mm to 6 mm), and three curved tubes (1.2 mm diameter) with varying orientations and bend radii. A mechanical fixture secured the 3D-printed target to a high-precision three-axis translation stage, ensuring the meta-imaging camera faced these microstructures in a top-down view (Fig. S13). Furthermore, both the checkerboard calibration pattern and the texture board pattern were printed on identical 50×50×3 mm substrates. This design allowed all components – the checkerboard, texture board, and 3D-printed target – to be mounted on the same fixture, establishing a shared world coordinate system (Fig. S13). This configuration enabled precise translation of the entire assembly along the Z-axis (optical axis) via the translation stage (Fig. S13). Thus, by positioning the checkerboard and texture board at different known depths, we acquired the precise 3D coordinates of checkerboard corner points and texture board features across depth planes. This facilitated joint disparity calibration and aberration correction, ultimately permitting the projection of the 3D-printed target's coordinates into the scanning light-field camera coordinate system to obtain ground-truth depth maps. Finally, utilizing the disparity-calibrated and aberration-corrected scanning light-field images, we estimated the depth of the miniature 3D-printed structures using the pre-trained FMNet.

In addition to the objects with sharp discontinuities shown in Fig. 3, we further conducted a depth estimation experiment on a continuously varying depth target (a 3D-printed flower) at an observation distance of 0.1 m, and the results are presented in Fig. S14. These results indicate that our method also maintains good accuracy in smoothly varying depth regions (0.61 mm at 0.1 m), slightly lower than the accuracy at sharp discontinuities (0.24 mm at 0.1 m). This small degradation may be attributed to the lack of surface texture, which makes depth estimation more challenging. Nevertheless, our method still markedly outperforms traditional light-field imaging

and MDS without aberration correction, consistent with the results on the 3D-printed posts in Fig. 3. These analyses confirm that the quantitative and qualitative results are consistent and mutually supportive.

Section S7: Long-range depth estimation

For long-range depth estimation, we developed a telescopic light-field imaging system (Fig. S15a) comprising a QHY600Pro camera with Sony IMX455 BSI CMOS sensor (3.76 μm pixel pitch, 36×24 mm sensor area, 9600×6400 effective pixels). The integrated microlens array features 0.10152 mm lenslet pitch and 3.90852 mm focal length (F/38.5). The optical chain combines a Celestron C14HD XLT telescope (356 mm aperture, 3910 mm focal length, F/11, StarBright XLT coating) with a custom relay module (Fig. S15b) matching F-numbers between components. The relay system maintains imaging resolution while providing 3.5× magnification, converting F/11 input to F/38.5 output.

Complementary depth measurements employ a Neuvition Titan M1-R long-range LiDAR system (600 m maximum range, 2 cm depth accuracy, 0.01° angular resolution, Fig. S15c). Limited LiDAR field-of-view necessitated three acquisition passes with subsequent point cloud stitching for full-scene coverage (Fig. 4b). Given the absence of LiDAR measurements in specific scenarios (Fig. 4c), we performed additional calibration using a high-precision laser range finder (UNI-T LM1000G) through manual feature point selection to establish the disparity-to-depth mapping.

For the long-range imaging system, we did not employ mechanical scanning. Since turbulence exerts a more severe impact on mechanical scanning and leads to greater depth reconstruction errors. Our implementation addresses this by using the Turbulence-Induced Scanning (TIS)[38] algorithm prior to multi-view depth estimation to enhance the spatial sampling rate of the sub-aperture images and to efficiently reduce the spatial inconsistency across sub-aperture views induced by turbulence

aberrations. For static scenes, temporal fusion further suppresses residual spatial inconsistencies by averaging spatially aligned depth estimates across multiple frames. In this way, high-resolution imaging and turbulence-resilient depth sensing can be achieved without mechanical scanning.

Section S8: Virtual scanning

The scanning light-field imaging system achieves mechanical 2D scanning through high-precision oscillations of a two-axis piezoelectric galvo mirror, inherently operating as a time-sequential acquisition system. This temporal nature imposes fundamental limitations on imaging efficiency, constrained by both mirror scanning speed and per-position exposure requirements, particularly hindering depth perception of moving targets. To address this limitation, we implement virtual scanning light-field imaging technology [36] (Fig. 5a) that computationally reconstructs high-throughput multi-view light-field images from single-shot acquisitions, bypassing mechanical scanning.

Virtual scanning technology employs a deep neural network to learn the mapping from low-resolution non-scanned light-field images to their scanned counterparts, originally developed for microscopy applications[36]. While microlens-based light-field systems inherently sacrifice spatial resolution for angular information, the sub-aperture views contain latent high-frequency components that can be recovered through optimized network architectures.

We adapt the Virtual Scanning Network (Vs-Net) [36] for macroscopic imaging by retraining with our experimental dataset. The network learns to reconstruct 4×4 scanned light-field images (16 scanning positions) from single central-scan-position inputs. Training employs the ADAM optimizer with initial learning rate 2×10^{-4}, halved every 100 epochs over 400 total iterations. Random 80×80 patches are

extracted from training images for network input. The PyTorch implementation runs on an NVIDIA RTX 3090 GPU with batch size 2.

Virtual scanning is fundamentally a high-resolution light field reconstruction algorithm. Therefore, artifacts may occur when reconstructing certain high-frequency image details. For instance, in the dense grid-like texture shown in Fig. S16, virtual scanning can introduce reconstruction errors that inevitably lead to inaccuracies in subsequent depth estimation. However, for the majority of scenes, virtual scanning can reconstruct high-throughput multi-view images with quality comparable to that of mechanical scanning. As shown in Fig. 5b, the performance degradation caused by virtual scanning is very limited relative to mechanical scanning, yet the gain it offers is high-throughput depth sensing of dynamic scenes, which cannot be achieved by mechanical scanning alone. Our video depth estimation results and additional visual comparisons further support the effectiveness of the virtual scanning modality.

Section S9: Video depth temporal continuity enhancement

The virtual scanning light-field imaging framework enables continuous video depth estimation, though inherent temporal inconsistency arises from our frame-wise disparity estimation network's single-frame optimization nature. This manifests as minor depth fluctuations around ground truth values across consecutive frames - a common challenge in monocular depth estimation. To address this, we implement and fine-tune Wang et al.'s Neural Video Depth Stabilizer ($NVDS^{+}$) [39] , which processes current and three preceding frames (images + depth estimates) to generate temporally filtered outputs. While the original $NVDS^{+}$ improves temporal coherence, its pre-trained implementation causes excessive smoothing that degrades depth details (Fig. S17b). We therefore develop a domain-specific fine-tuning strategy leveraging DA-v2's video depth predictions that inherently maintain temporal consistency and detail preservation.

The fine-tuning implementation involves synthetic training pair generation through DA-v2-derived depth maps with inherent temporal stability, modified by adding spatially smooth temporal noise. This noise generation process begins with low-resolution random noise (1%-4% disparity range) that undergoes bicubic upsampling to DA-v2 resolution before superposition onto original DA-v2 depths, creating temporally inconsistent training inputs (Fig. S17a).

The composite loss function combines three components: depth constraints using standard L_1 loss between predictions and DA-v2 depths, surface normal regularization through cosine similarity, and temporal consistency enforcement. The temporal term employs optical flow estimates from GMFlow [50] to warp previous frame depths (D_{n-1}) to current frame coordinates ($\widehat{D}_{n-1}$), comparing them with current estimates (D_n) through occlusion-aware masking ($O_{n\Rightarrow n-1}$) [51,52] with M pixels. The complete loss expression is:

$$\mathcal{L}_{NVDS^{+}} = \mathcal{L}_D + 0.002\mathcal{L}_N + 0.0015\mathcal{L}_T \tag{11}$$

with the temporal term defined as:

$$\mathcal{L}_T = \sum_{x=1}^{M} O_{n\Rightarrow n-1}(x)\left|D_n(x) - \widehat{D}_{n-1}(x)\right| \tag{12}$$

Training utilizes over 200 frames from four video sequences, processed as 320×320 random patches through ADAM optimization (initial learning rate 5×10^{-5}, halved every 40 epochs over 100 total epochs). The PyTorch implementation runs on an NVIDIA RTX 3090 GPU with batch size 1. This fine-tuned NVDS^{+} maintains detailed depth resolution compared to original pre-trained NVDS^{+} (Fig. S17b), demonstrating the effectiveness of fine-tune strategy.

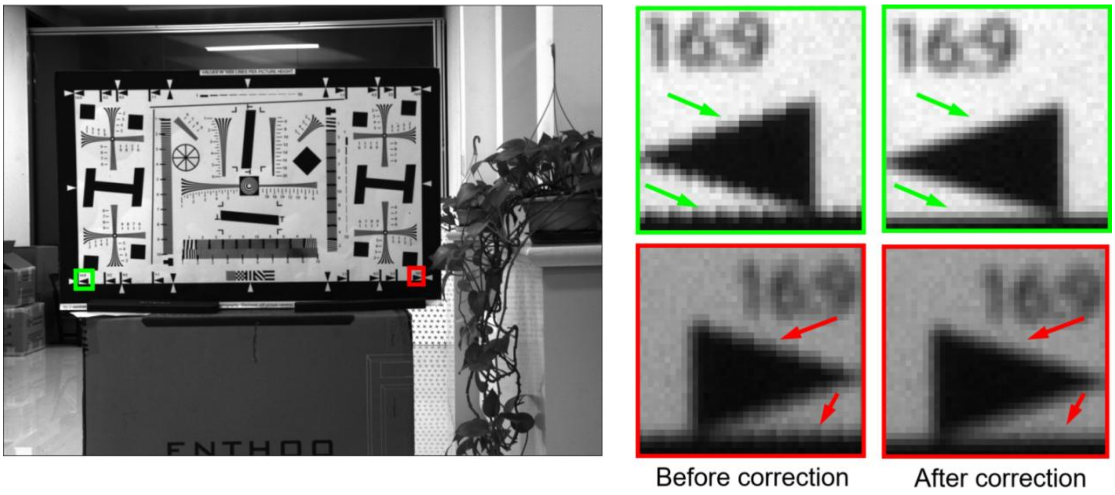

Figure S1| Comparison of light-field image reconstruction results before and after correction with the proposed compensation algorithm.

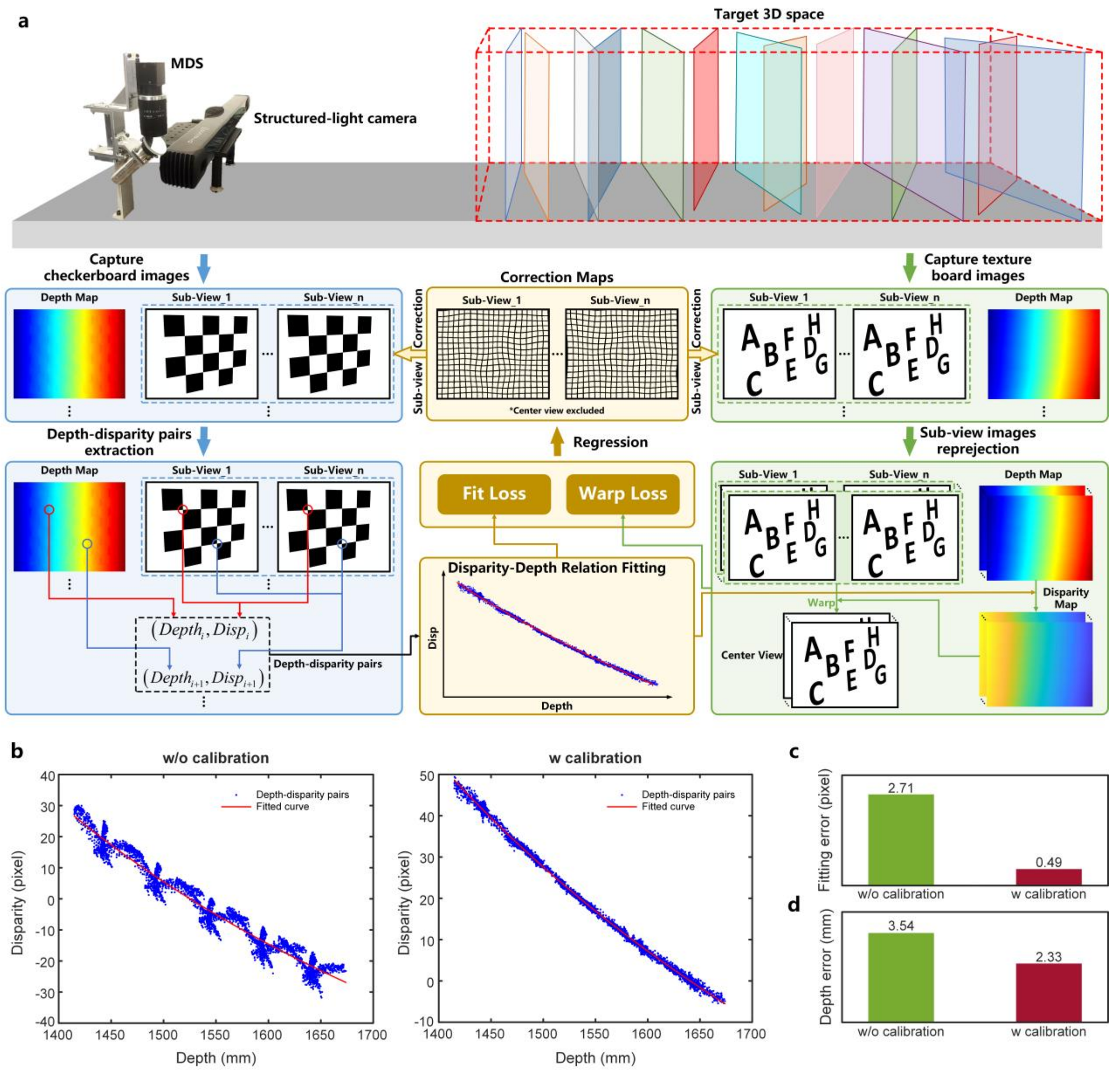

Figure S2| Joint aberration-disparity calibration algorithm. (a) The framework of proposed joint aberration-disparity calibration algorithm. (b) Disparity-depth fitting error of the depth meta-imaging system with and without joint calibration. (c) The average disparity-depth fitting error of the MDS before and after joint calibration. (d) The average depth estimation error of the MDS before and after joint calibration.

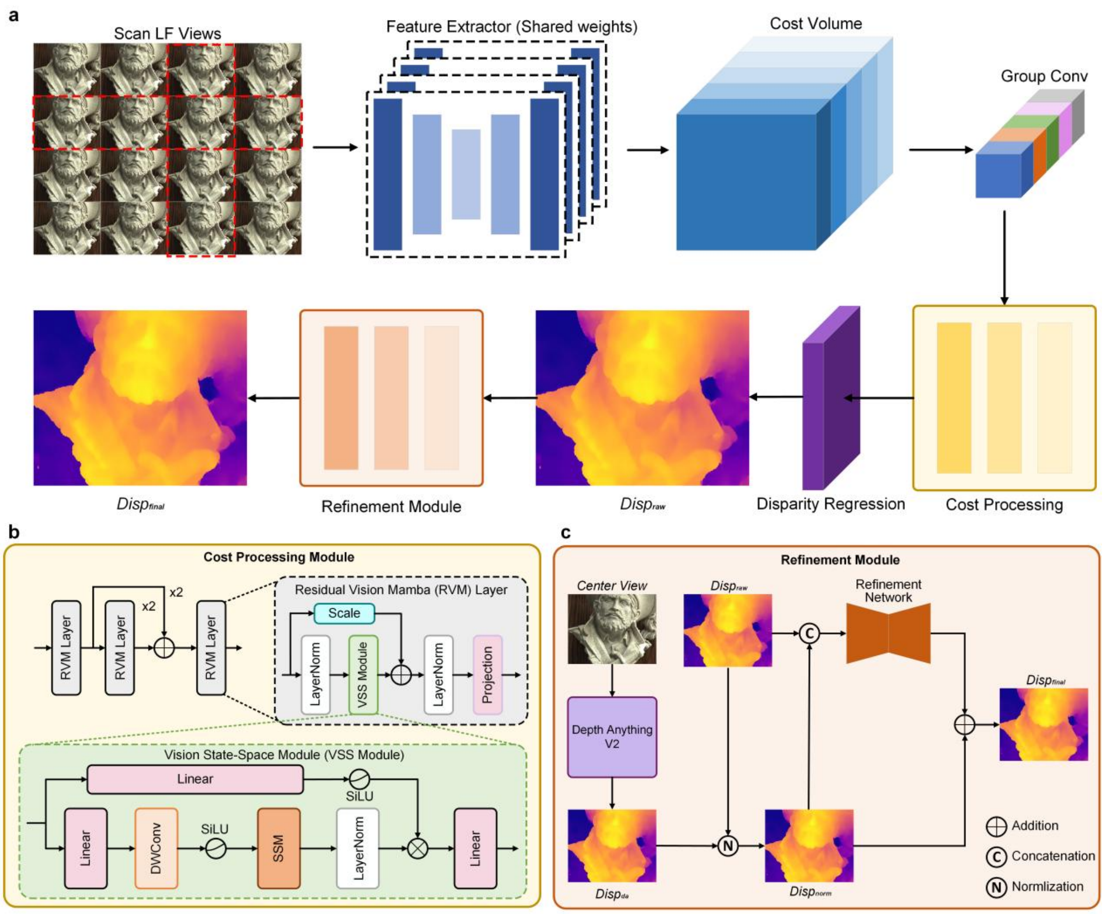

d

Module	Params (M)	GFLOPs	Depth error (mm)
3D dila conv	3.98	300.97	2.64
ViT	1.63	116.36	2.52
Mamba	**1.05**	**112.09**	**2.33**

Figure S3| Overview of depth vision Foundation model guided Mamba network (FMNet) architecture. (a) The overall structure of the FMNet. (b) Cost volume processing module based on vision-mamba structure. (c) Disparity refinement module based on depth vision foundation model guidance. (d) Comparative analyses of the Mamba network against other architectures—including Vision Transformers (ViT) and 3D convolutional networks — reveal that Mamba achieves superior depth reconstruction accuracy with significantly fewer parameters and lower computational overhead, highlighting its distinct advantages in efficiency and performance.

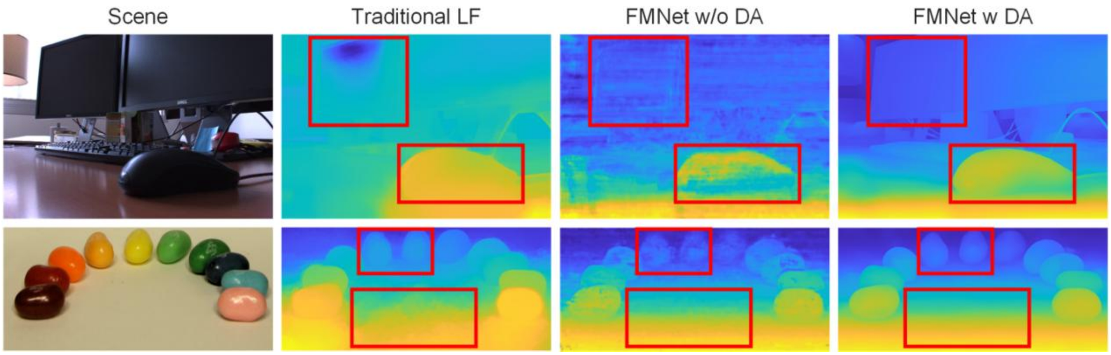

Figure S4| Disparity estimation results on weak-texture scenes. We evaluated traditional light-field disparity estimation algorithms, the proposed FMNet, and its variant without DA assistance on two weak-texture scenes: one captured by a Lytro camera [53] and the other by a gantry camera [54].

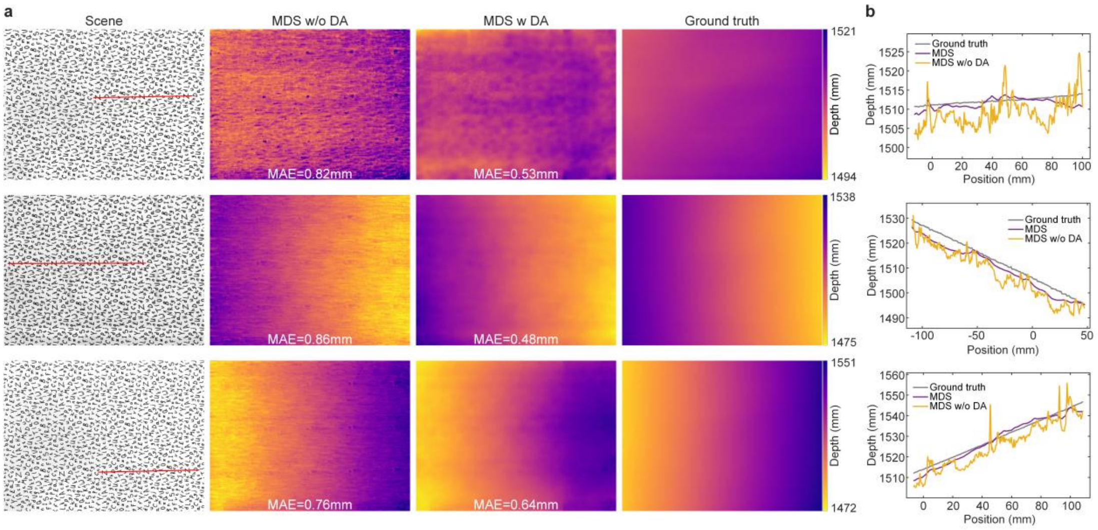

Figure S5| Depth measurement results on objects with varying albedo. (a) Depth estimation results and mean absolute depth estimation errors of MDS with and without the assistance of the DA vision foundation model. (b) Comparison of depth profile curves along the red line in (a) obtained by different methods.

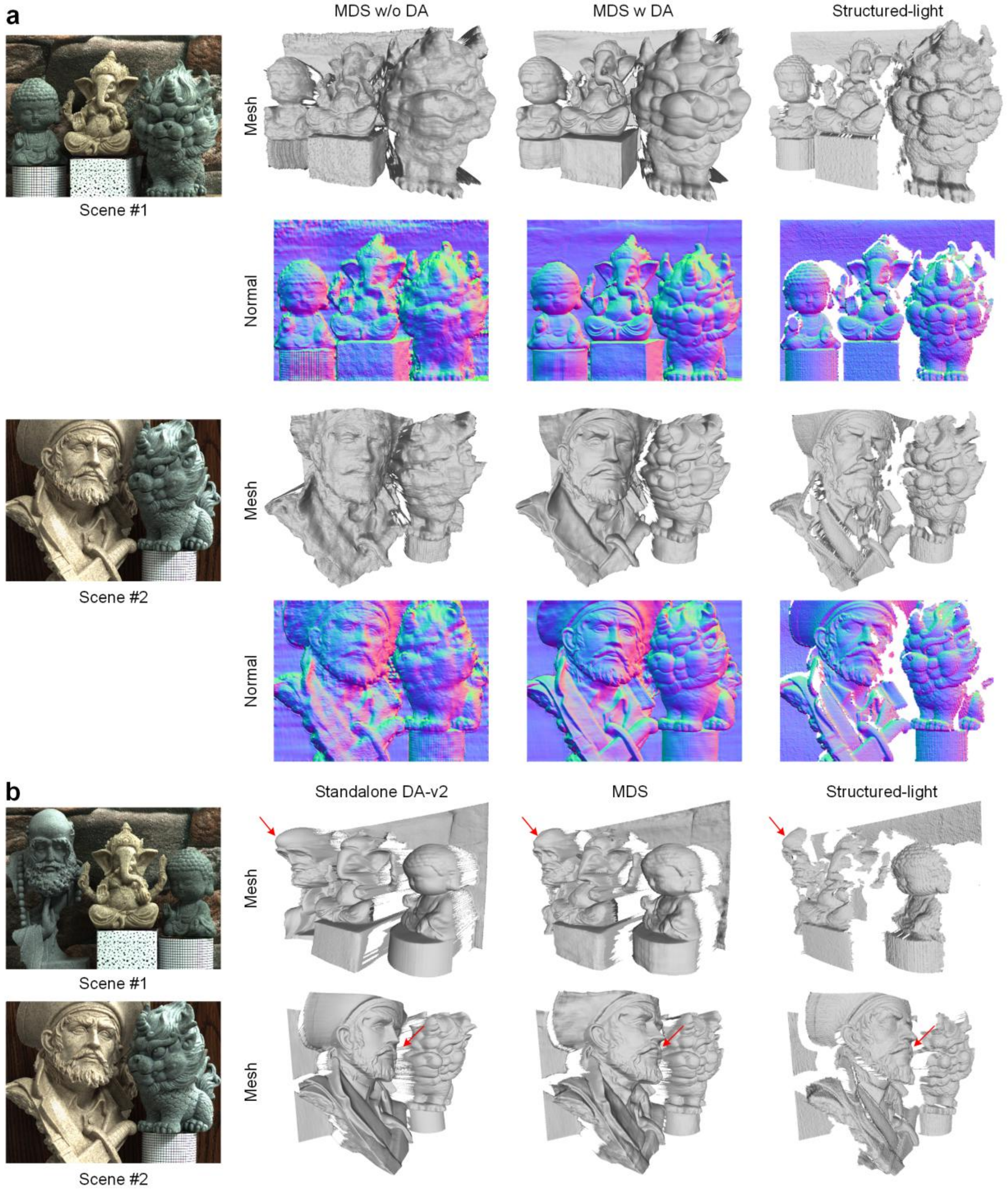

Figure S6| The effectiveness of the depth vision foundation model. (a) Comparison of mesh and normal results obtained by MDS with and without DA guidance. (b) Comparison of mesh and normal results obtained by MDS and standalone DA-v2. For both (a) and (b), the corresponding results of structured-light camera are provided for reference.

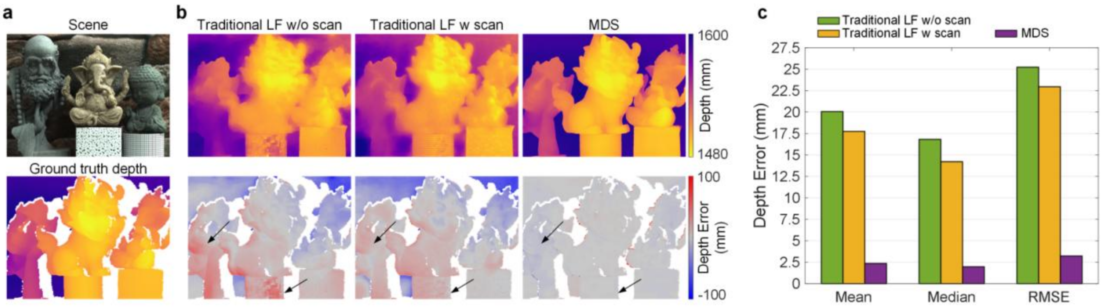

Figure S7| Evaluation of traditional light-field depth estimation algorithms on scanning light-field data. (a) One test scene and its ground truth depth. (b) Depth estimation results and corresponding error maps obtained by different methods. (c) Mean depth estimation errors of different methods on the test data.

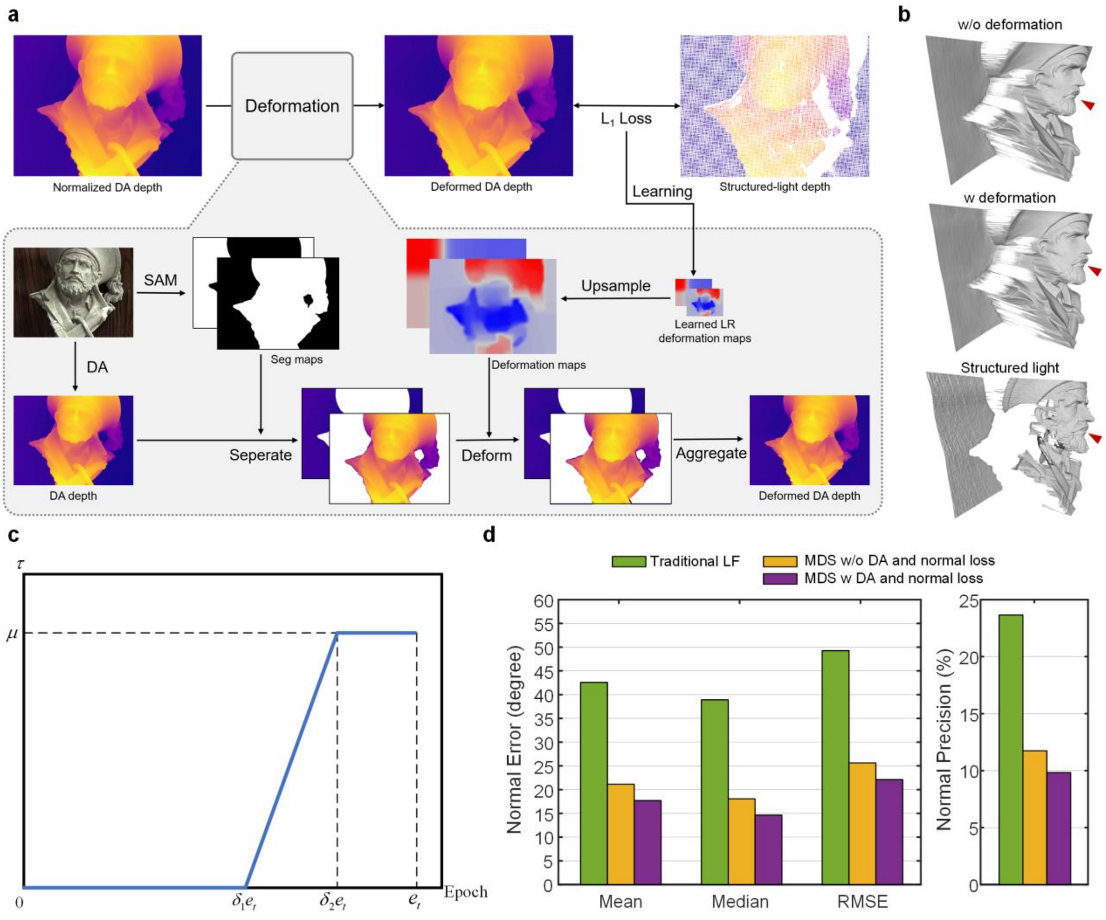

Figure S8| Training strategy utilizing depth foundation model predictions to constrain the proposed FMNet. (a) The deformation method of DA depth prediction to align with structured-light depth results. (b) Comparison of mesh results obtained by DA with and without deformation. (c) Modification scheme of normal loss term with DA-v2's prediction. (d) Surface normal estimation error and precision of traditional light-field imaging versus MDS under different setups. It should be noted that while we employ surface normals derived from structured-light depth measurements as ground truth, these represent locally averaged normal vectors due to the significantly lower spatial resolution of structured-light systems compared to meta-imaging. Given this resolution limitation, our comparative analysis of surface normal reconstruction performance across different algorithms is necessarily constrained to this framework.

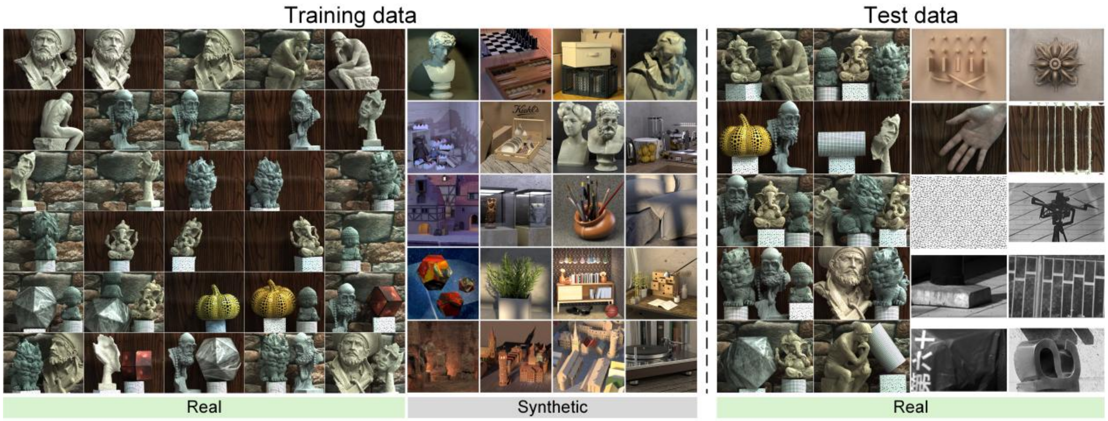

Figure S9| Data used for model training and test.

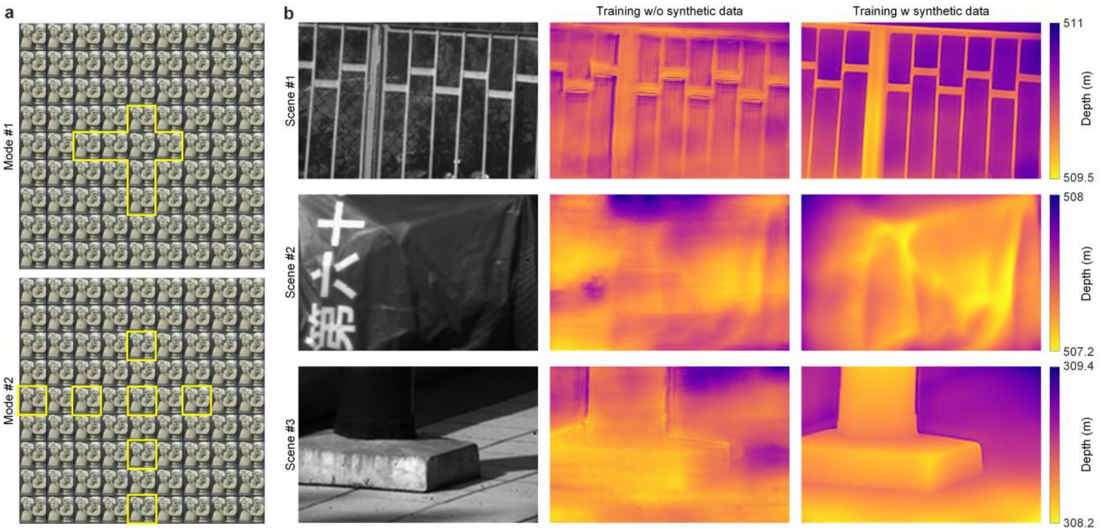

Figure S10| Influence of using synthetic data for training. (a) View selection modes of synthetic light-field data. (b) Comparison of depth estimation performance of the MDS with and without using synthetic training data.

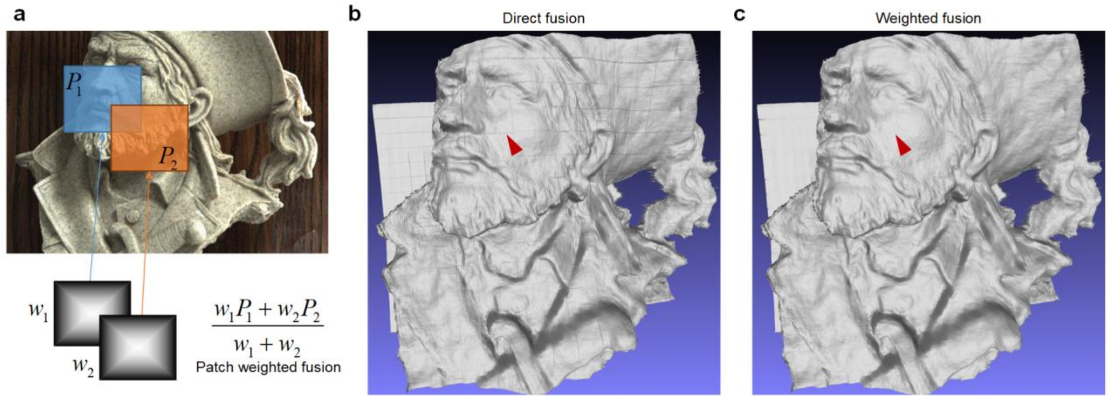

Figure S11| Weighted patch fusion for high-throughput depth reconstruction. (a) Depth patch weighted fusion method for full-resolution depth reconstruction. (b) Mesh results obtained by direct patch fusion. (c) Mesh results obtained by weighted patch fusion.

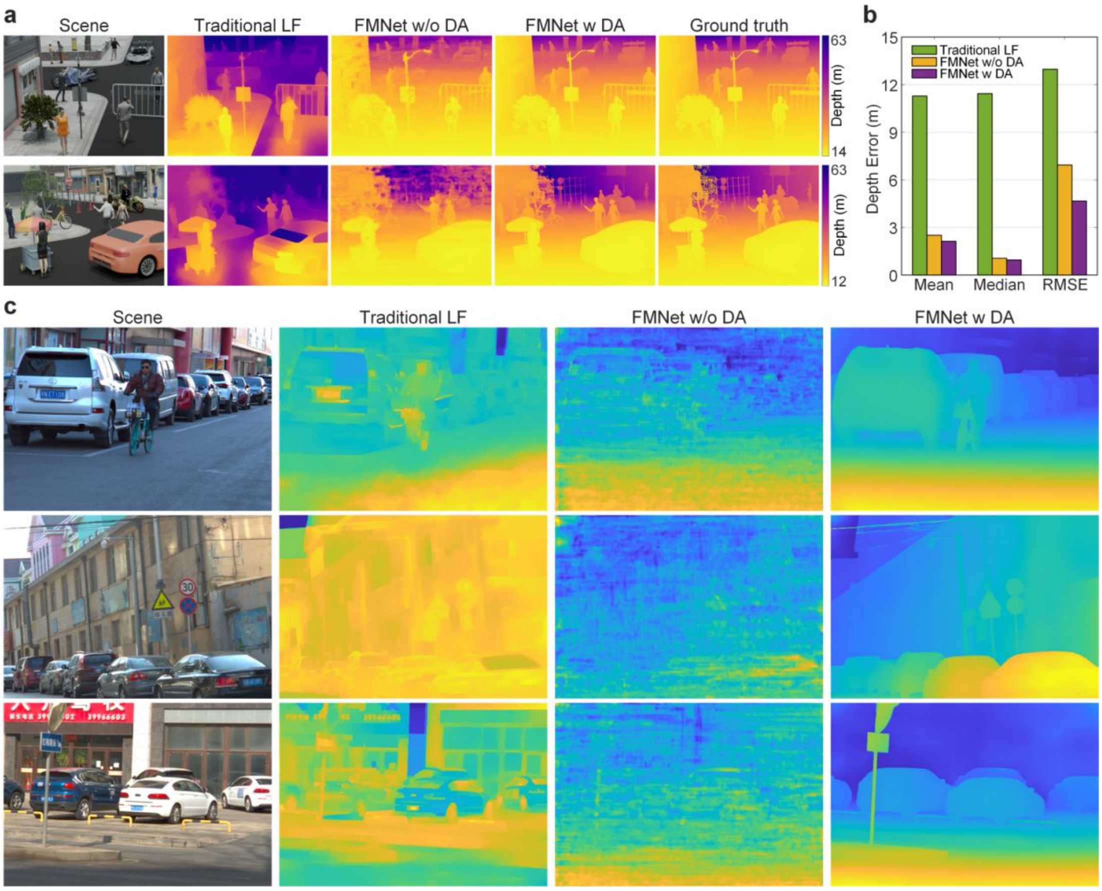

Figure S12| Generalization test of the proposed FMNet on urban autonomous driving scenes. (a) Depth estimation results of different algorithms on synthetic light-field data of urban road scenes from UrbanLF val set. (b) Depth estimation errors of different algorithms on synthetic light-field data of urban road scenes from UrbanLF val set. (c) Disparity estimation results of different algorithms on real light-field data of urban road scenes from UrbanLF real dataset.

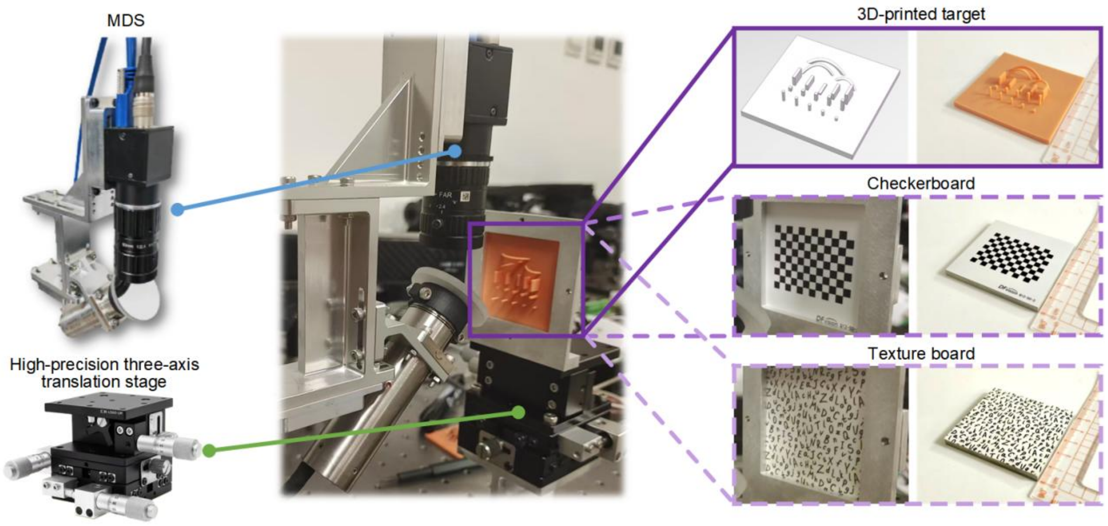

Figure S13| Experimental setup for close-range depth estimation. For close-range depth estimation, we employed the MDS depicted in Fig. 1b, substituting the primary lens with an f=12 mm, F/2 lens to achieve an appropriate field of view at a 10 cm working distance. A mechanical fixture secured the 3D-printed target to a high-precision three-axis translation stage, ensuring the meta-imaging camera faced these microstructures in a top-down view. Furthermore, both the checkerboard calibration pattern and the texture board pattern were printed on identical 50×50×3 mm substrates. This design allowed all components – the checkerboard, texture board, and 3D-printed target – to be mounted on the same fixture, establishing a shared world coordinate system. This configuration enabled precise translation of the entire assembly along the Z-axis (optical axis) via the translation stage. Thus, by positioning the checkerboard and texture board at different known depths, we acquired the precise 3D coordinates of checkerboard corner points and texture board features across depth planes.

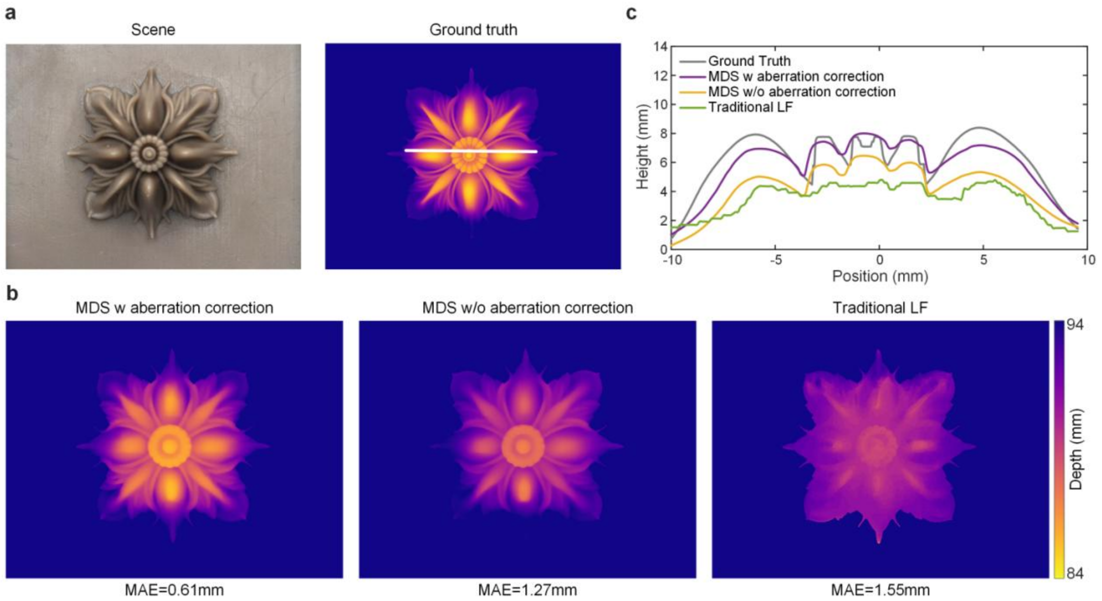

Figure S14| Depth estimation experiment on a continuously varying depth target (a 3D-printed flower) at an observation distance of 0.1 m. (a) Test object and its ground truth depth map. (b) Depth estimation results of different methods. (c) Comparison of height profile curves along the white line in (a) obtained by different methods.

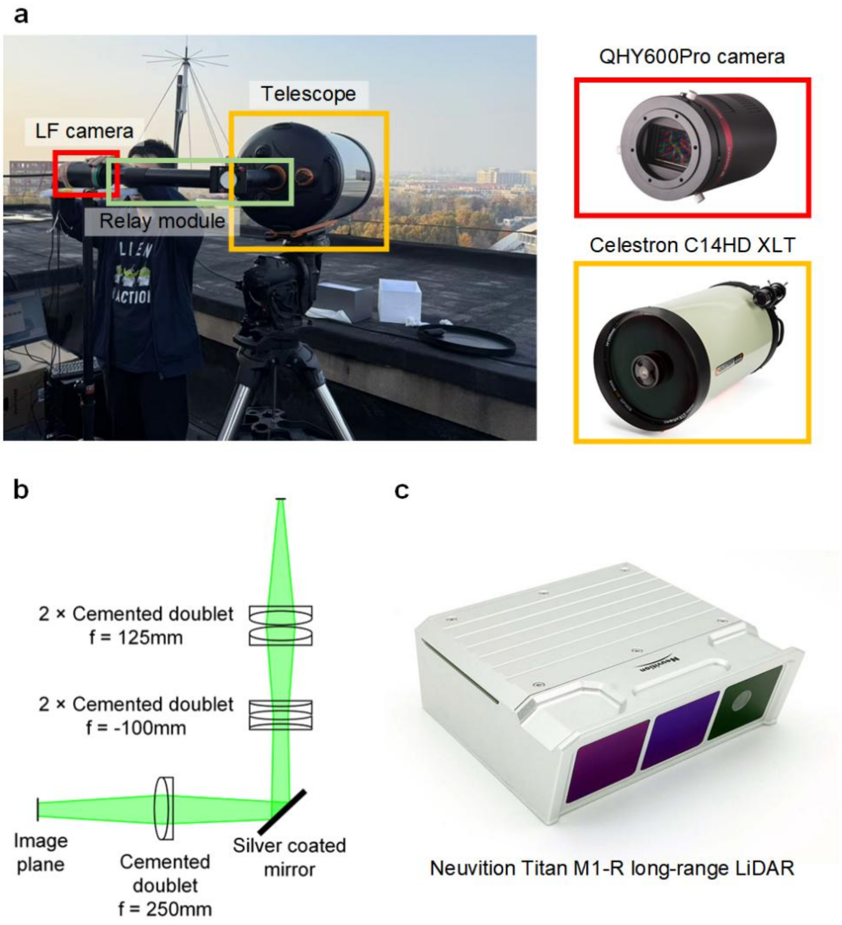

Figure S15| Long range light-field imaging system. (a) A telescopic light-field imaging system comprising LF camera, a relay module, and a telescope. (b) The optical schematic of the relay system used in telescopic setup. (c) A Neuvition Titan M1-R long-range LiDAR system with 600m maximum range, 2cm depth accuracy, and 0.01° angular resolution.

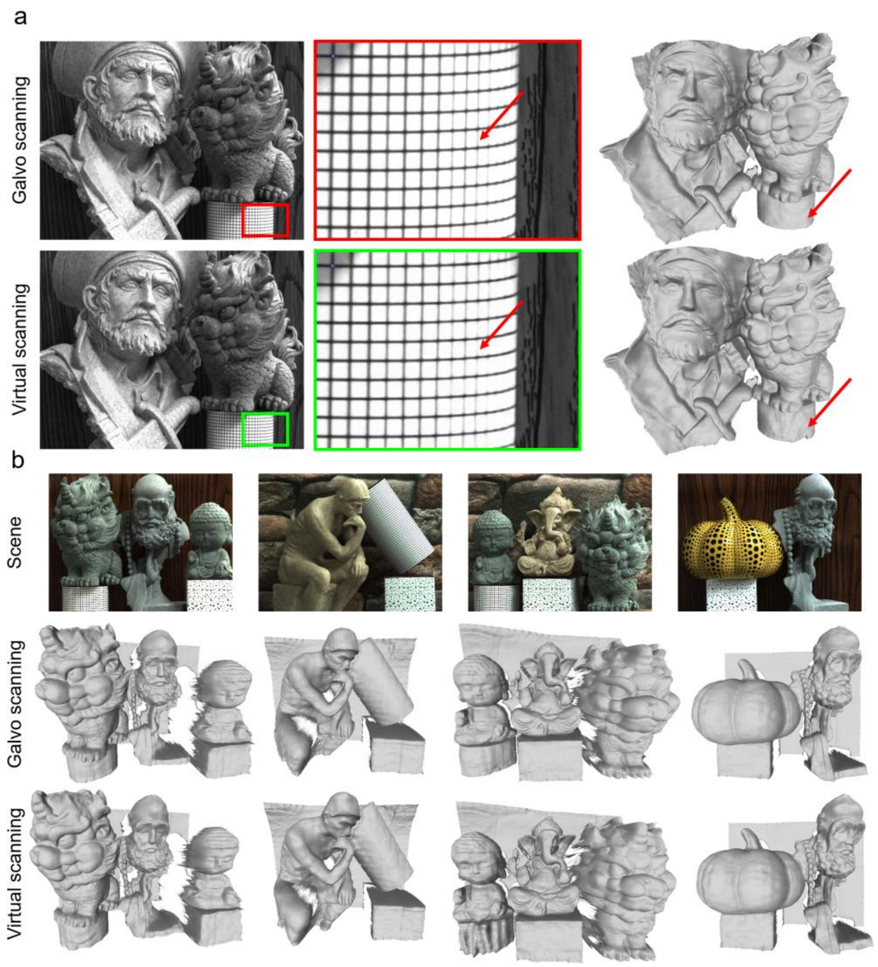

Figure S16| Comparison of light-field image reconstruction quality and depth estimation results between virtual scanning and galvo scanning. (a) Comparison of light-field image reconstruction quality between virtual scanning and galvo scanning. (b) Comparison of light-field depth reconstruction results between virtual scanning and galvo scanning.

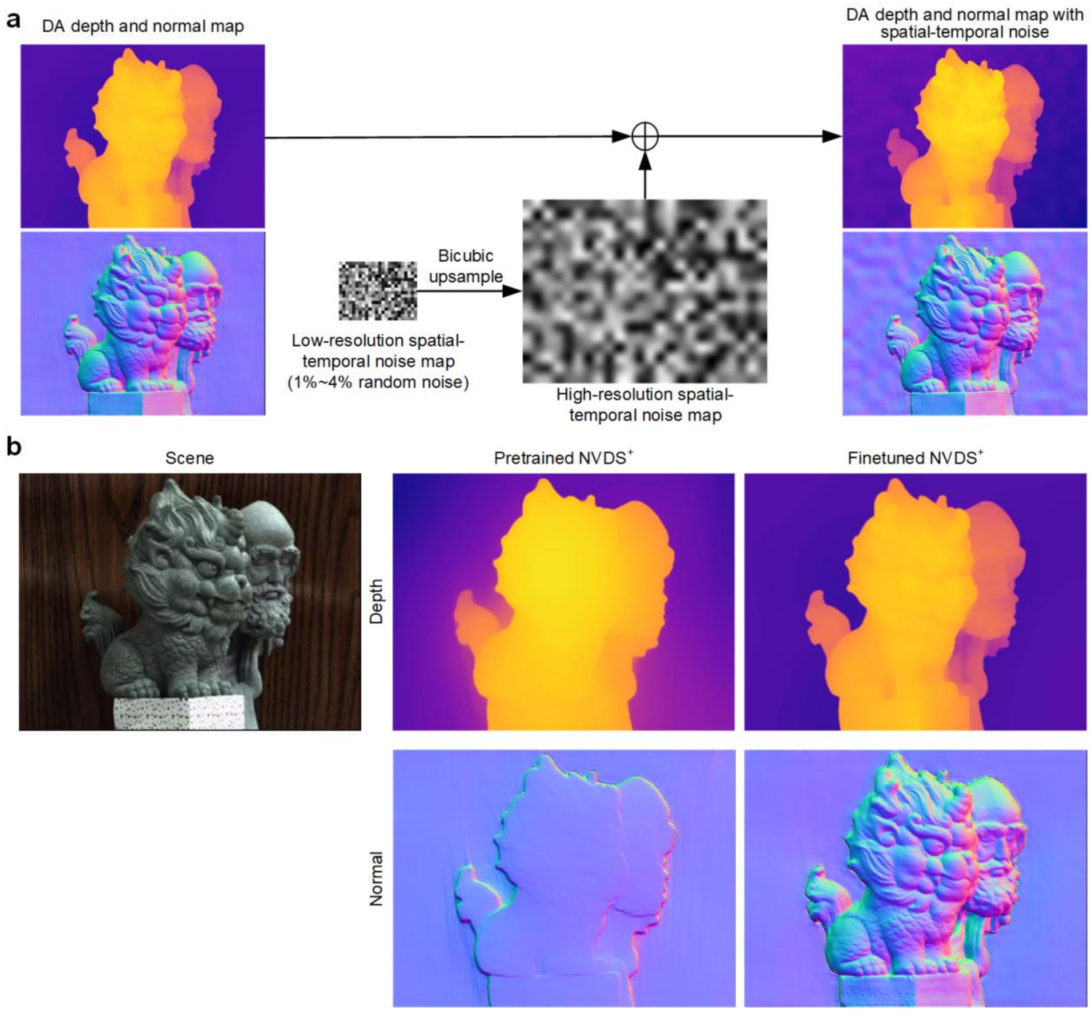

Figure S17| Fine-tuning scheme of NVDS+. (a) A schematic diagram synthesizing a depth estimation sequence containing spatiotemporal random noise based on DA-v2 estimated depth results. (b) Comparison of depth and normal results obtained by pretrained and finetuned NVDS+.

References

40. He, K., Zhang, X., Ren, S., & Sun, J. Spatial pyramid pooling in deep convolutional networks for visual recognition. IEEE Trans. Pattern Anal. Mach. Intell. 37, 1904-1916 (2015).
41. Gu, A., & Dao, T. Mamba: Linear-time sequence modeling with selective state spaces. arXiv preprint arXiv 2312, 00752 (2023).
42. Li, Z., Liu, X., Drenkow, N., Ding, A., Creighton, F. X., Taylor, R. H., & Unberath, M. Revisiting stereo depth estimation from a sequence-to-sequence perspective with transformers. In Proc. IEEE International Conference on Computer Vision (ICCV) 6197-6206 (IEEE, 2021).
43. Ba, Y., Gilbert, A., Wang, F., Yang, J., Chen, R., Wang, Y., ... & Kadambi, A. Deep shape from polarization. In Proc. European Conference on Computer Vision (ECCV) 554-571 (2020).
44. Kirillov, A., Mintun, E., Ravi, N., Mao, H., Rolland, C., Gustafson, L., ... & Girshick, R. Segment anything. In Proc. IEEE International Conference on Computer Vision (ICCV) 4015-4026 (IEEE, 2023).
45. Honauer, K., Johannsen, O., Kondermann, D., & Goldluecke, B. A dataset and evaluation methodology for depth estimation on 4D light fields. In Proc. Asian Conference on Computer Vision (ACCV) 19-34 (Springer, 2017).
46. Shin, C., Jeon, H. G., Yoon, Y., Kweon, I. S., & Kim, S. J. Epinet: A fully-convolutional neural network using epipolar geometry for depth from light field images. In Proc. IEEE Conference on Computer Vision and Pattern Recognition (CVPR) 4748-4757 (IEEE, 2018).
47. Wang, Y., Wang, L., Liang, Z., Yang, J., An, W., & Guo, Y. Occlusion-aware cost constructor for light field depth estimation. In Proc. IEEE Conference on Computer Vision and Pattern Recognition (CVPR) 19809-19818 (IEEE, 2022).
48. Wang, Y., Wang, L., Wu, G., Yang, J., An, W., Yu, J., & Guo, Y. Disentangling light fields for super-resolution and disparity estimation. IEEE Trans. Pattern Anal. Mach. Intell. 45, 425-443 (2022).
49. Sheng, H., Cong, R., Yang, D., Chen, R., Wang, S., & Cui, Z. UrbanLF: A comprehensive light field dataset for semantic segmentation of urban scenes. IEEE Trans. Circuits Syst. Video Technol. 32, 7880-7893 (2022).
50. Xu, H., Zhang, J., Cai, J., Rezatofighi, H., & Tao, D. GMFlow: Learning optical flow via global matching. In Proc. IEEE Conference on Computer Vision and Pattern Recognition (CVPR) 8121-8130 (IEEE, 2022).
51. Wang, Y., Pan, Z., Li, X., Cao, Z., Xian, K., & Zhang, J. Less is more: Consistent video depth estimation with masked frames modeling. In Proc. ACM International Conference on Multimedia 6347-6358 (ACM, 2022).
52. Cao, Y., Li, Y., Zhang, H., Ren, C., & Liu, Y. Learning structure affinity for video depth estimation. In Proc. ACM International Conference on Multimedia 190-198 (ACM, 2021).
53. INRIA Lytro image dataset, http://clim.inria.fr/research/LowRank2/datasets/Dataset_LytroIllum.zip
54. Sunder Raj, A., Lowney, M., Shah, R., & Wetzstein, G. The (new) stanford light field archive, (Online, 2016).
